# Electronic dynamics created at conical intersections and its dephasing in aqueous solution

**DOI:** 10.1038/s41567-024-02703-w

**Published:** 2024-11-27

**Authors:** Yi-Ping Chang, Tadas Balciunas, Zhong Yin, Marin Sapunar, Bruno N. C. Tenorio, Alexander C. Paul, Shota Tsuru, Henrik Koch, Jean-Pierre Wolf, Sonia Coriani, Hans Jakob Wörner

**Affiliations:** 1 GAP–Biophotonics, Université de Genève, Geneva, Switzerland; 2 European XFEL, Schenefeld, Germany; 3 Laboratory of Physical Chemistry, ETH Zürich, Zurich, Switzerland; 4 International Center for Synchrotron Radiation Innovation Smart, Tohoku University, Sendai, Japan; 5 Division of Physical Chemistry, Ruđer Bošković Institute, Zagreb, Croatia; 6 Department of Chemistry, Technical University of Denmark, Kongens Lyngby, Denmark; 7 Instituto Madrileño de Estudios Avanzados en Nanociencia, IMDEA-Nanociencia, Madrid, Spain; 8 Department of Chemistry, Norwegian University of Science and Technology, Trondheim, Norway; 9 Lehrstuhl für Theoretische Chemie, Ruhr-Universität Bochum, Bochum, Germany; 10 RIKEN Center for Computational Science, RIKEN, Kobe, Japan

**Keywords:** Atomic and molecular interactions with photons, Electronic structure of atoms and molecules

## Abstract

A dynamical rearrangement in the electronic structure of a molecule can be driven by different phenomena, including nuclear motion, electronic coherence or electron correlation. Recording such electronic dynamics and identifying its fate in an aqueous solution has remained a challenge. Here, we reveal the electronic dynamics induced by electronic relaxation through conical intersections in both isolated and solvated pyrazine molecules using X-ray spectroscopy. We show that the ensuing created dynamics corresponds to a cyclic rearrangement of the electronic structure around the aromatic ring. Furthermore, we found that such electronic dynamics were entirely suppressed when pyrazine was dissolved in water. Our observations confirm that conical intersections can create electronic dynamics that are not directly excited by the pump pulse and that aqueous solvation can dephase them in less than 40 fs. These results have implications for the investigation of electronic dynamics created during light-induced molecular dynamics and shed light on their susceptibility to aqueous solvation.

## Abstract (web summary)

Tracking ultrafast electronic changes in molecules is challenging, especially in liquids. An X-ray spectroscopy study in pyrazine now shows electronic dynamics created at conical intersections that are rapidly suppressed when the molecule is in water.

## Main

Electronic dynamics is the central concept underlying several emerging research fields ranging from attosecond spectroscopy^1,2^ and attochemistry^3–6^ to quantum biology^7,8^. Despite its importance, identifying electronic dynamics and distinguishing it from purely vibrational dynamics has remained a major challenge and has caused numerous controversies. For excited-state dynamics, in particular, the distinction between electronic and purely vibrational dynamics often remains out of reach because of the strong coupling between the two types of dynamics in the vicinity of a conical intersection or because of insufficient contrast in the sensitivity of the probe to electronic versus vibrational dynamics. Additionally, an important question for the viability and broader impact of the above-mentioned research areas is the lifetime of electronic dynamics under ambient conditions in the liquid phase, which implies that there is a rapidly fluctuating environment^9^. Moreover, many technologically and fundamentally important processes, especially in chemistry and biology, take place in water, so that exploiting electronic dynamics in such processes^10^ necessitates a method that can trace and disambiguate them in aqueous solutions. Although two-dimensional spectroscopy in the optical domain has, in principle, this capability, the assignment and interpretation of the observed signals often remain sufficiently ambiguous that attempts to do so have created controversies, for example, regarding the nature and lifetime of electronic versus vibrational dynamics observed in light-harvesting systems^7,8^.

Here, we describe two experimental breakthroughs: (1) the observation of electronic and vibrational dynamics corresponding to a circular rearrangement of the electronic structure created by conical intersection dynamics and (2) the sub-40 fs dephasing induced by aqueous solvation. This was achieved by directly comparing the dynamics of ultraviolet (UV)-excited pyrazine in the gas phase and in aqueous solution with an experimental scheme that unites element specificity, site selectivity and the transparency of water with the required time resolution. This scheme combines one-photon excitation in the UV domain with a soft-X-ray probe^11–14^ to cover the entire water window^15^. The interpretation of our results is supported by the latest advances in quantum-chemical calculations for X-ray absorption spectroscopy (XAS) coupled to non-adiabatic dynamical simulations that also include the effects of solvation. These calculations enabled an unambiguous assignment of the transient spectral features observed in the experimental spectra.

The heteroaromatic pyrazine molecule (C_4_H_4_N_2_), a paradigmatic system for both theoretical and experimental studies of electronically non-adiabatic dynamics, serves as a demonstration of the opportunities opened by our work. The gas-phase measurements indeed show that the electronic relaxation of the initially photo-excited ^1^B_2u_(ππ^*^) state (historically referred to as S_2_) through conical intersections induces electronic and vibrational dynamics involving the ^1^B_3u_(nπ^*^, known as S_1_) and the ^1^A_u_(nπ^*^) states. Our gas-phase results, moreover, resolve a decades-old controversy between ever-evolving dynamics simulations^16–18^ that eventually converged on the prediction of oscillatory population flow^19–24^ and experiments that have always negated them. Specifically, time-resolved photoelectron spectroscopy has revealed neither the population of the ^1^A_u_ state nor the quasi-periodic electronic dynamics^25–29^, whereas carbon K-edge transient absorption has suggested that the ^1^A_u_ state is populated in ~200 fs—up to ten times slower than the theoretical predictions—but has not found any oscillations^13^. As shown here, the key to resolving this controversy was combining single-photon excitation with transient absorption spectroscopy at the nitrogen K-edge at ~405 eV. The unprecedented capability of nitrogen K-edge spectroscopy allowed us not only to confirm the predicted oscillatory population flow but also to show that it corresponds to a cyclic rearrangement of the electronic structure around the aromatic ring of pyrazine.

Beyond confirming the possibility of creating electronic dynamics at conical intersections and resolving the important controversy regarding the electronic-relaxation pathway of the isolated pyrazine molecule, our work additionally revealed the effect of aqueous solvation on the paradigmatic dynamics of this molecule. Specifically, we found that the electronic and vibrational dynamics are completely dephased by solvation within 40 fs. A comparison with dynamical calculations indeed confirmed that solvation strongly dampens the observed dynamics but also revealed that two explicit water molecules combined with a continuum-solvation model are insufficient to capture fully the dephasing observed in the solution-phase experiments.

**Fig. 1:**
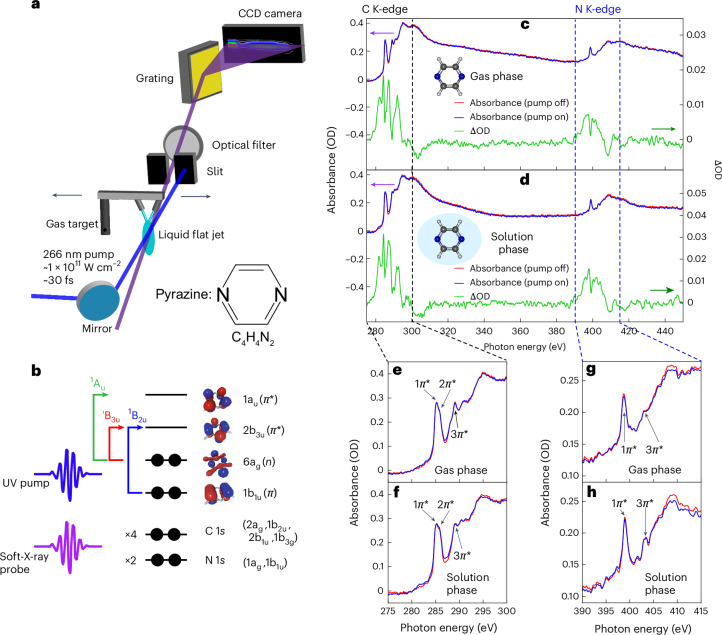
Overview of the experimental methods and results. **a**, Schematic of the experimental set-up with both gas and solution flat-jet targets. **b**, Molecular-orbital diagram of the photo-excitation of pyrazine. Arrows indicate the main transition character of the valence excited states: ^1^B_2u_(ππ^*^), ^1^B_3u_(nπ^*^) and ^1^A_u_(nπ^*^). **c**,**d**, XAS spectra and time-averaged differential absorbances (over 150 fs) covering the water window from the carbon to nitrogen K-edges for the gas phase (**c**) and the solution phase (**d**). No energy shift in the ground-state pyrazine (static measurement) at the carbon pre-edge peak (285.3 and 285.8 eV) was observed between the solution and gas phases. The nitrogen pre-edge peak was 398.7 eV in the gas phase compared to 398.9 eV in the solution phase, so that there was an energy shift of about 0.2 eV. **e**,**f**, Enlargement of the carbon K-edge for the gas phase (**e**) and the solution phase (**f**). **g**,**h**, Enlargement of the nitrogen K-edge for the gas phase (**g**) and the solution phase (**h**).

Figure 1 provides an overview of the experimental set-up (Fig. 1a), a diagram of the relevant orbitals of pyrazine (Fig. 1b) and the XAS spectra (Fig. 1c–h). The experiments were performed by photo-exciting pyrazine with a 30 fs pump pulse centred at 266 nm and focused to an intensity of ~1 × 10^11^ W cm^−^^2^. It was then probed with a soft-X-ray supercontinuum extending beyond 450 eV obtained from high-harmonic generation of a ~12 fs pulse centred at 1.8 μm in helium. Here, we demonstrate the unique capability of soft-X-ray spectroscopy in the water window to directly compare the dynamics of the same molecules in the gas and solution phases. For this purpose, a dedicated target system was constructed that allowed us to rapidly switch between a gas cell delivering an effusive beam of pyrazine vapour and a liquid flat jet running a 5 M aqueous solution of pyrazine. More details of the experimental set-up are given in the Supplementary Information.

Figure 1c,d shows the static XAS spectra of gaseous pyrazine and a 5 M aqueous pyrazine solution from the carbon to the nitrogen K-edges (red curves), respectively. The time-averaged transient absorption of the excited sample is shown by the blue curves. The differential absorbance spectrum (ΔOD) is shown in green. Magnified portions of the absorption spectra at each edge for both phases are shown in Fig. 1e–h.

At the carbon K-edge, a strong pre-edge absorption feature consisting of two subpeaks is observed. In both gaseous and aqueous samples, the two pre-edge peaks are at 285.3 and 285.8 eV and can be assigned to the C 1*s* → 1π^*^ [2a_g_ → 2b_3u_] and 1*s* → 2π^*^ [1b_3g_ → 1a_u_] transitions (Supplementary Figs. 11 and 14). The two final core excited states have B_3u_ symmetry. In the gas phase, a shoulder at ~288.2 eV and a peak at ~289.1 eV can be observed, corresponding to the 1*s* → σ^*^/Rydberg and 1*s* → 3π^*^ [2b_1u_ → 2b_2g_] transitions, respectively. In the solution phase, the shoulder at 288.2 eV cannot be resolved, whereas the peak at ~289.0 eV is still present with a small energy shift, but the same assignments as in the gas phase apply.

At the nitrogen K-edge, a single strong pre-edge absorption peak can be observed at 398.7 and 398.9 eV, for the gaseous and aqueous samples, respectively. This corresponds to the N 1*s* → 1π^*^ [1a_g_ → 2b_3u_] transition and indicates an energy shift of +0.2 eV from the gas to the solution phase. In the gas phase, a peak at 402.9 eV corresponding to the N 1*s* → 3π^*^ [1b_1u_ → 2b_2g_] transition can be observed. In the solution phase, a broad peak was seen at ~403.3 eV.

The experimental spectra were calibrated by aligning the experimental gaseous pyrazine carbon and nitrogen pre-edge peaks with synchrotron measurements^30^. In both the gas and solution phases, the ΔOD spectra exhibited increased absorption up to 10 eV below and above the pre-edge. Most of the pre-edge absorption features originated from allowed transitions into valence vacancies created by the pump pulse. The rest of the pre-edge absorption features, as well as the above-pre-edge ones, shared core-excitation characters with those of transitions from the ground state.

**Fig. 2:**
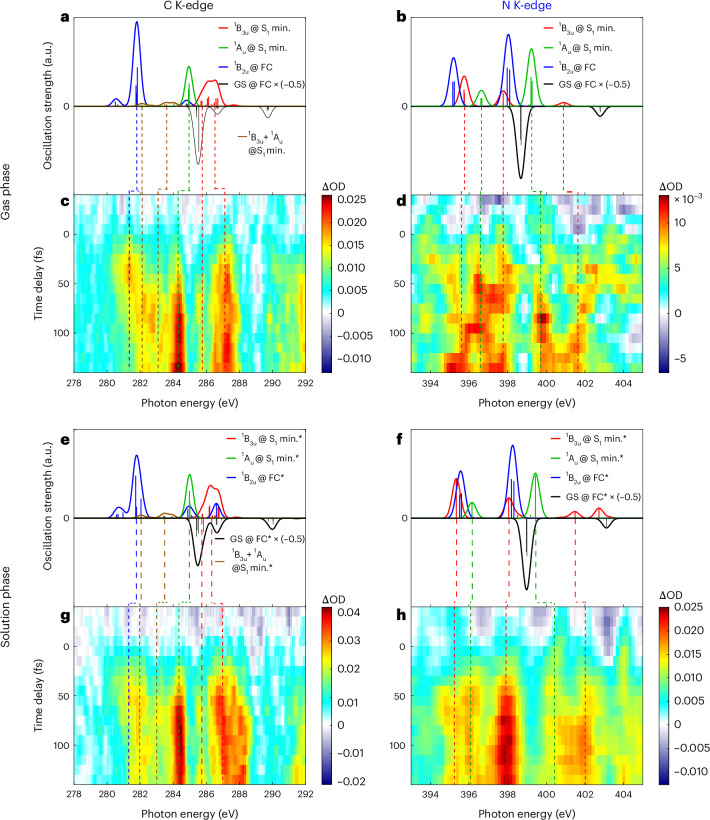
Time-resolved differential absorbance spectra of pyrazine in the gas and aqueous-solution phases. **a**,**e**, Carbon K-edge excited-state XAS spectra calculated at the RASPT2/RAS2(10e,8o) level for both the FC and the relaxed geometry for the first valence excited state (S_1_), with (**e**) and without (**a**) the PCM. Asterisks indicate cases with the PCM. GS, ground state; min., minimum. **b**,**f**, As in **a** and **e** at the nitrogen K-edge, calculated with (**f**) and without (**b**) the PCM. **c**,**d**, Time-resolved differential absorbance spectra at the carbon (**c**) and nitrogen (**d**) K-edges of gaseous pyrazine. **g**,**h**, Time-resolved differential absorbance spectra at the carbon (**g**) and nitrogen (**h**) K-edges of 5 M aqueous pyrazine.

Figure 2 shows the time-resolved ΔOD spectra of gaseous pyrazine (Fig. 2c,d) and 5 M aqueous pyrazine solution (Fig. 2g,h), recorded over a ~150 fs time window with 10 fs steps.

At the carbon K-edge in the gas phase (Fig. 2c), a positive absorption band at 281.4 eV was observed in the first ~50 fs, after which it disappeared and was replaced by another positive band centred at 282.2 eV, which was broad in the initial ~50–100 fs and then narrowed. Concurrently, another positive band at 284.2 eV emerged, which had a longer rise time than the previous two bands. At the pre-edge, there was a weak band at ~285.7 eV. Above the pre-edge, a positive band at 287.2 eV was observed rising earlier than the 284.2 eV band. A comparison of the 284.2 and 287.2 eV bands revealed that they oscillated weakly out of phase (Supplementary Fig. 1).

At the carbon K-edge in water solution (Fig. 2g), a broad positive absorption band centred at ~281.2 eV was also observed in the first ~50 fs, after which it disappeared. At the same time, another broad positive band centred at ~282 eV also emerged, such that the band shift from ~281.2 to 282 eV was less clear compared to the gas-phase signal. Concurrently, another positive band at 284.4 eV emerged and peaked in intensity at ~100 fs before gradually declining. At the pre-edge, there was a weak band at ~285.6 eV. Above the pre-edge, a positive band at 286.8 eV was observed rising at nearly the same time as the 284.4 eV band, before peaking at ~60 fs and subsequently declining in intensity. No quantum beats were observed in either the 284.4 or the 286.8 eV band.

To assign the bands, both the carbon and nitrogen K-edge excited-state XAS spectra were calculated at the RASPT2/RAS2(10e,8o) level for both the Franck–Condon (FC) geometry and the minimum of the first valence excited singlet state (S_1_), the latter obtained with and without the polarizable continuum model (PCM) for gaseous and aqueous pyrazine, respectively. The relaxed geometries were obtained at the CASPT2 level. All RAS/CAS calculations were performed using OpenMOLCAS^31^. Additionally, coupled cluster calculations including perturbative triples (CC3)^32,33^ were performed. The carbon and nitrogen K-edge excited-state XAS spectra were calculated at the CC3 level of theory for the gas-phase FC and the S_1_ minimum geometry using the eT program^34,35^. The spectra and more details about the calculations can be found in the Supplementary Information. Note that in the gas phase, the potential-energy minimum of the S_1_ state has a mixed configuration, with contributions from both nπ^*^ states. This is reflected in the simulated spectrum, which shows peaks corresponding to those seen for both the ^1^B_3u_ and ^1^A_u_ states for the FC geometry.

At the carbon K-edge in the gas phase, based on the calculated spectra shown in Fig. 2a and the schematics in Supplementary Fig. 9, we assigned the ~281.4 eV experimental band to the 281.7 eV C 1*s* → *π* transition from the ^1^B_2u_(ππ^*^) state for the FC geometry, the ~282.2 eV band to the 282.1 eV C 1*s* → *n* transitions from both the ^1^B_3u_(nπ^*^) and ^1^A_u_(nπ^*^) states for the S_1_ minimum geometry (which are highly overlapping in this region; Supplementary Fig. 12), the broad band around 283 eV also to the calculated C 1*s* → *n* transitions at around 283.8 eV from both ^1^B_3u_(nπ^*^) and ^1^A_u_(nπ^*^), the 284.2 eV band to the 284.2 eV C 1*s* → π^*^ transition from the ^1^A_u_(nπ^*^) state for the S_1_ minimum geometry, the ~285.7 eV band to the 285.9 eV C 1*s* → π^*^ transition from the ^1^B_3u_(nπ^*^) state for the S_1_ minimum geometry, and the ~287.2 eV band to the 287.2 eV C 1*s* → π^*^ transition from the ^1^B_3u_(nπ^*^) state for the S_1_ minimum geometry.

At the carbon K-edge in the solution phase, based on the calculated spectra shown in Fig. 2e, we assigned the ~281.2 eV band to the 281.8 eV C 1*s* → *π* transition from the ^1^B_2u_(ππ^*^) state for the FC geometry, the ~281.9 eV band to the 281.9 eV C 1*s* → *n* transition from both the ^1^B_3u_(nπ^*^) and ^1^A_u_(nπ^*^) states for the S_1_ minimum geometry (Supplementary Fig. 13), the weak and broad band around 283 eV to the calculated 1*s* → *n* transitions at around 283.6 eV from both ^1^B_3u_(nπ^*^) and ^1^A_u_(nπ^*^), the ~284.4 eV band mainly to the 284.7 eV C 1*s* → π^*^ transition from the ^1^A_u_(nπ^*^) for the S_1_ minimum geometry, the ~285.6 eV band to the 285.7 eV C 1*s* → π^*^ transition from the ^1^B_3u_(nπ^*^) state for the S_1_ minimum geometry, and the ~286.8 eV band to the 286.8 eV C 1*s* → π^*^ transition from the ^1^B_3u_(nπ^*^) state for the S_1_ minimum geometry.

Looking now at the nitrogen K-edge in the gas phase (Fig. 2d), for which the pre-edge was at 398.8 eV, a broad positive absorption band from ~393.6 to 395.2 eV was observed in the first ~50 fs before disappearing. Concurrently, positive absorption bands centred at ~395.3, 396.4 and 397.6 eV emerged and showed clear modulations in intensity. Looking above the pre-edge, two bands at 399.5 and 401 eV emerged at the same time as the others. These two above-pre-edge bands showed clearer intensity modulations that were out of phase with each other.

At the nitrogen K-edge in the solution phase (Fig. 2h), for which the pre-edge was at 398.9 eV, a weak positive absorption band at ~396 eV appeared initially before broadening into a broad band from ~394 to 396.2 eV at ~50 fs. After ~50 fs, this broad band narrowed into two bands at ~395.2 and ~396.3 eV. Moreover, a strong absorption band at 397.8 eV emerged from time zero and plateaued around 100 fs. Above the edge, a weak band at ~400.3 eV started emerging from time zero and merged into a broad peak from ~400 to 402 eV at ~70 fs.

At the nitrogen K-edge in the gas phase, based on the calculated spectra shown in Fig. 2b (and the schematics in Supplementary Fig. 8), we assigned the broad ~393.6 to 395.2 eV band to the 395.2 eV N 1*s* → *π* [1b_1u_ → 1b_2g_ in the limit of *D*_2*h*_ symmetry] transition from the ^1^B_2u_(ππ^*^) state for the FC geometry, the ~395.3 eV band to the 395.5 eV N 1*s* → *n* transition of the ^1^B_3u_(nπ^*^) state for the S_1_ minimum geometry [1b_1u_ → 6a_g_ in the *D*_2*h*_ limit, to a final core state of symmetry B_2g_], the ~396.4 eV band to the 396.4 eV N 1*s* → *n* transition of the ^1^A_u_(nπ^*^) state for the S_1_ minimum geometry [also 1b_1u_ → 6a_g_ in the *D*_2*h*_ limit, but to a final core state of symmetry B_1g_], the ~397.6 eV band to the 397.6 eV N 1*s* → π^*^ [1a_g_ → 2b_3*u*_(1π^*^) in the *D*_2*h*_ limit, final core state A_g_] transition from the ^1^B_3u_(nπ^*^) state for the S_1_ minimum geometry, the ~399.5 eV band to the 399.2 eV N 1*s* → π^*^ transition from the ^1^A_u_(nπ^*^) state [also 1a_g_ → 2b_3u_(1π^*^) in the *D*_2*h*_ limit, but to a final B_3g_ core state] for the S_1_ minimum geometry, and the ~401 eV band to the 401 eV N 1*s* → π^*^ [1b_1u_ → 2b_2g_(3π^*^) in the *D*_2*h*_ limit] transition from the ^1^B_3u_(nπ^*^) state for the S_1_ minimum geometry.

At the nitrogen K-edge in the solution phase, based on the calculated spectra shown in Fig. 2f, we assigned the initial 396 eV band to a mix of the 396.1 eV N 1*s* → *π* transition from the ^1^B_2u_(ππ^*^) state for the FC geometry and the 396.2 eV N 1*s* → *n* transition of the ^1^A_u_(nπ^*^) state for the S_1_ minimum geometry. The ~395.2 eV band was assigned to the 395.2 eV N 1*s* → *n* transition from the ^1^B_3u_(nπ^*^) state, the ~397.8 eV band to the 397.7 eV N 1*s* → π^*^ transition from the ^1^B_3u_(nπ^*^) state for the S_1_ minimum geometry, the ~400.3 eV band to the 399.4 eV N 1*s* → π^*^ transition from the ^1^A_u_(nπ^*^) state for the S_1_ minimum geometry, and the ~402 eV band to the 401.5 eV N 1*s* → π^*^ (3π^*^ in *D*_2*h*_) transition from the ^1^B_3u_(nπ^*^) state for the S_1_ minimum geometry.

**Fig. 3:**
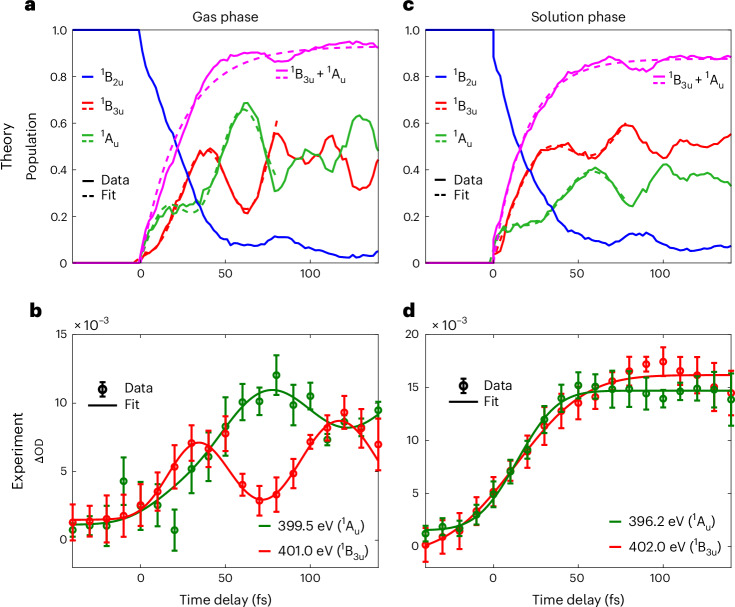
Observation of electronic and vibrational dynamics in gas-phase pyrazine and their dephasing in aqueous solution. **a**,**c**, Populations of the diabatic ^1^B_2u_(ππ^*^), ^1^B_3u_(nπ^*^) and ^1^A_u_(nπ^*^) states based on FSSH trajectories for the gas phase (**a**) and solution phase (**c**). **b**, Time-dependent ΔOD of gaseous pyrazine for the bands centred at 399.5 and 401.0 eV. According to calculation results shown in Fig. 2b, these bands are associated with the ^1^A_u_(nπ^*^) and ^1^B_3u_(nπ^*^) characters, respectively. **d**, Time-dependent ΔOD of 5 M aqueous pyrazine for the bands centred at 396.2 and 402 eV. According to calculation results shown in Fig. 2f, these bands are associated with the ^1^A_u_(nπ^*^) character and ^1^B_3u_(nπ^*^) characters, respectively. The error bars indicate the measured standard deviations over 12 and nine sets of scans for the gas- and solution-phase measurements, respectively.

The time dependence of these nitrogen K-edge differential absorbance bands over 150 fs is shown in Fig. 3, together with the calculated time-dependent populations (Fig. 3a). These diabatic populations were obtained by projecting wavefunctions calculated along trajectories using the fewest-switches surface-hopping (FSSH) method onto diabatic electronic wavefunctions defined at the ground-state minimum geometry^36^. These deep, antiphased quantum beats indicate efficient population transfer between the ^1^A_u_ and ^1^B_3u_ states. The calculated spectral intensities corresponding to these FSSH calculations display the same trends as the population dynamics shown in Supplementary Fig. 21, which provides further details of these calculations. In gaseous pyrazine above the nitrogen pre-edge, the 399.5 and 401 eV bands (Fig. 3b) were fitted with the product of a step function and a cosine convoluted with a Gaussian of 30 fs full-width at half-maximum. From peak to peak, the 401 eV band rises earlier than the 399.5 eV band by ~40 fs. It has two local maxima at ~40 and ~120 fs and a local minimum at ~70 fs, with a period of ~80 fs. The 399.5 eV band has local maxima at ~80 and ~150 fs and a local minimum at ~120 fs, with a period of ~70 fs. Given that the 399.5 and 401 eV bands are associated with dominant ^1^A_u_(nπ^*^) and ^1^B_3u_(nπ^*^) characters, respectively, the observed ΔOD quantum beats in the two bands reflect the electronic dynamics involving these two states. When fitted with a sigmoidal function convoluted with a cosine, both the 399.5 and 401 eV bands have a rise time of 50 ± 30 fs with a time constant of 30 ± 15 fs and are nearly π out of phase with respect to each other.

The experimentally observed quantum beat agrees well with the predictions of the gas-phase calculations shown in Fig. 3a. The red and green curves for ^1^B_3u_(nπ^*^) and ^1^A_u_(nπ^*^), respectively, were fitted with the product of a rising exponential and a cosine function, which revealed that they were close to π out of phase with respect to each other. The purple curve for the summed populations of the ^1^B_3u_(nπ^*^) and ^1^A_u_(nπ^*^) states was fitted with a rising exponential function, yielding a time constant of 24.0 ± 1.2 fs.

At the nitrogen K-edge in aqueous pyrazine (Fig. 3d), the 396.2 and 402 eV bands, which are associated with the ^1^A_u_(nπ^*^) and ^1^B_3u_(nπ^*^) characters, respectively, do not display any visible quantum beats. These data were, therefore, fitted with sigmoidal functions. The 396.2 and 402 eV bands have rise times of 50 ± 20 fs and 80 ± 20 fs, respectively. As a caveat, note that the observation of pure ^1^A_u_(nπ^*^)/^1^B_3u_(nπ^*^) dynamics was prevented by the overlap of three peaks at 396.2, 396.1 and 395.2 eV, which were assigned to transitions from the ^1^A_u_(nπ^*^), ^1^B_2u_(ππ^*^) and ^1^B_3u_(nπ^*^) states, respectively. Nevertheless, the absence of quantum beats in the 402.0 eV band is a reliable indicator of the lack of quantum beats in the solution phase. The calculation results shown in Fig. 3c, which include two explicit water molecules and a conductor-like screening model (COSMO), do indeed predict a smaller contrast (roughly by a factor of two) of the quantum beat compared to the isolated-molecule calculations (Fig. 3a). The complete suppression of the quantum beats in the experiment must, therefore, involve other mechanisms, such as decoherence and dissipation, that go beyond those included in our simulations. The calculated rise time of the summed populations in the solution phase is shorter than in the gas-phase calculations and amounts to 18.8 ± 0.6 fs.

We now discuss these new insights into the creation of electronic and vibrational dynamics in isolated and solvated pyrazine molecules in the light of the theoretical results. After photo-excitation to the ^1^B_2u_ state, the molecular system moves away from the FC geometry in <30 fs following the slope of the excited-state potential-energy surface. This initial motion stabilizes all three excited states with respect to the ground state and shifts the corresponding XAS peaks. Subsequently, we found that the positions of the bands remains fairly constant and, especially at the nitrogen K-edge, they are most easily assigned based on those calculated at the minimum of the S_1_ state. Our experimental results obtained at both edges and in both phases of matter clearly show that both the ^1^A_u_(nπ^*^) and the ^1^B_3u_(nπ^*^) states are populated within a few tens of femtoseconds, creating electronic and vibrational dynamics in the gas phase that are suppressed in the solution phase. The spectra obtained in the solution phase are analogous to those from the gas phase but with some notable differences. Peaks corresponding to the ^1^A_u_(nπ^*^) state seem to rise earlier than in the gas phase, which is probably due to the ^1^A_u_/^1^B_2u_ conical intersection lying directly in the FC region as a consequence of the different solvation shifts of the two states.

**Fig. 4:**
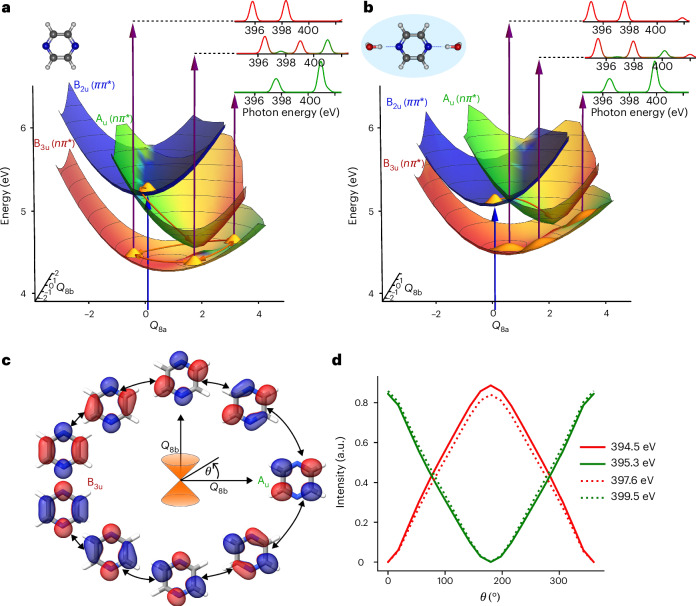
Wave-packet dynamics in isolated and solvated pyrazine molecules. **a**, Adiabatic potential-energy surfaces of the three lowest excited states of isolated pyrazine as a function of the *Q*_8a_ and *Q*_8b_ normal modes computed at the ADC(2)/aug-cc-pVDZ level of theory. Surfaces are coloured according to contributions of the ^1^B_3u_, ^1^A_u_ and ^1^B_2u_ electronic characters in the wavefunctions of the excited states. **b**, As in **a** but for solvated pyrazine calculated with two explicit water molecules hydrogen-bonded to the nitrogen atoms and COSMO. Details of the calculations are given in the Supplementary Information. **c**, Particle NTOs for the S_1_ state for geometries sampling a path around the conical intersection as indicated by arrows on the S_1_ surfaces in **a**. The colours of the isosurfaces encode the signs of the NTOs. **d**, Relative intensities of absorption bands characteristic of the ^1^A_u_ (green) and ^1^B_3u_ (red) states along a two-dimensional circular path spanned by the *Q*_8a_ and *Q*_8b_ normal modes encircling the ^1^B_3u_/^1^A_u_ conical intersection.

To rationalize these findings, Fig. 4 displays the potential-energy surfaces of the relevant electronic states of isolated (Fig. 4a) and solvated (Fig. 4b) pyrazine. We chose the *Q*_8a_ and *Q*_8b_ modes, as the ^1^A_u_/^1^B_3u_ conical intersection lies along the *Q*_8a_ mode and the *Q*_8b_ mode is responsible for the coupling between these two states^19^. This was further confirmed by the fact that population transfer was suppressed when the vibronic coupling mediated by the *Q*_8b_ mode was set to zero (Supplementary Fig. 21). The *Q*_8a_ and *Q*_8b_ modes, thus, represent the pair of tuning and coupling modes, respectively, that are most relevant to the ^1^A_u_/^1^B_3u_ dynamics. In the gas phase, the ^1^B_2u_/^1^A_u_ conical intersection is very close to the FC region, which explains the rapid population of both states. After crossing the ^1^A_u_/^1^B_3u_ conical intersection, electronic dynamics is created that involves both states in the gas phase and manifests itself in the observed quantum beats. The motion of the FSSH trajectories along these modes is shown in Supplementary Fig. 20 and further analysed in Supplementary Figs. 21 and 22 by running multi-configuration time-dependent Hartree calculations on the potential-energy surfaces of ref. ^19^.

The electronic dynamics underlying the observed quantum beats are illustrated in Fig. 4c, which shows the natural transition orbitals (NTOs) for the S_1_ state for geometries sampling a closed path around the ^1^B_3u_/^1^A_u_ conical intersection. By following the evolution of the NTOs, we can see their rotation around the aromatic ring of pyrazine, which is reminiscent of a ring current. Ring currents in molecules have attracted considerable interest in recent years (refs. ^37–39^ and references therein), but experimental evidence for such dynamics has been lacking. Although a unidirectional ring current cannot be generated in our experiment for symmetry reasons, the observed quantum beats can be interpreted as the consequence of a circular shift of the NTO around the aromatic ring. Our FSSH calculations, indeed, show that the excited-state trajectories can be grouped into two categories that encircle the ^1^B_3u_/^1^A_u_ conical intersection either clockwise or anticlockwise (Supplementary Fig. 20). The corresponding electronic dynamics are in excellent agreement with multi-configuration time-dependent Hartree calculations run on the same potential-energy surfaces (Supplementary Fig. 21). The multi-configuration time-dependent Hartree calculations themselves provide a consistent picture by showing that the excited-state wave packet splits into two components that encircle the conical intersection either clockwise or anticlockwise (Supplementary Fig. 22). Overall, these results show that the electronic dynamics observed in this work corresponds to a circular rearrangement of the electronic structure around the aromatic ring of the molecule.

Our calculations additionally clarify why the observed electronic dynamics are directly accessible at the nitrogen K-edge. Whereas the NTO of the ^1^B_3u_ state has a large amplitude on the nitrogen atoms, that of the ^1^A_u_ state has none, which leads to an intuitive interpretation for why the observed quantum beats are out of phase and best visible at the nitrogen K-edge, as illustrated in Fig. 4d. The intensities in Fig. 4d were calculated along a tight circular path around the conical intersection (*r* = 0.1 in dimensionless normal-mode coordinates), showing that they were, indeed, primarily sensitive to the electronic character. Finally, because of the geometric phase^40,41^, a closed loop around a conical intersection is expected to result in a reversal of the signs of both the electronic and the nuclear wavefunctions, which is reflected in the phase of the NTOs shown in Fig. 4c as well as in a node that appears in the nuclear wave packet after its two components have encircled the conical intersection in opposite directions^42^ (Supplementary Fig. 22).

Turning to the role of solvation, our calculations show that solvation stabilizes the ππ^*^ state, whereas it destabilizes both nπ^*^ states. This finding has been qualitatively reproduced for a microsolvation model^43^ (for details, see Supplementary Section 3.3) and is in agreement with the effects of solvation that were computationally found for nucleobases^44^. As Fig. 4 shows, the effects of solvation on pyrazine, thus, changed both the location of the conical intersections and their slopes. Solvation, indeed, moved the location of the ^1^B_2u_/^1^A_u_ conical intersection and led to a mixture of the two configuration characters near the FC point, which explains the earlier rise times of the corresponding bands in the solution phase. Comparing the effects of solvation on the two nπ^*^ states, we found that the ^1^A_u_ state was more destabilized by solvation than the ^1^B_3u_ state. This led to an energetic skew of the path on which the wave packet encircled the conical intersection, contributing to the dephasing of the quantum beats. However, as mentioned above, our solution-phase calculations (shown in Figs. 3b and 4b) only partially account for the effects of solvation by including two explicit water molecules, hydrogen-bonded to the nitrogen atoms of pyrazine, and COSMO. Importantly, this partial account of solvation was, nevertheless, sufficient to reduce the contrast of the quantum beats by a factor of two compared to the gas phase (Fig. 3a). Figure 3c identifies the differential solvation shifts as the probable origin of this partial dephasing. As our experimental results show no discernible quantum beats, we concluded that other mechanisms of decoherence and dissipation must have caused the observed complete dephasing on a timescale shorter than half of the 70–80 fs period of the observed quantum beats.

Our work shows that conical intersection dynamics can create electronic dynamics that correspond to a large-amplitude rearrangement of the electronic structure and simultaneously resolves a decades-old controversy regarding the electronic-relaxation pathway and dynamics of pyrazine. Such electronic dynamics could be exploited for efficient long-range charge transfer^45^ or for inducing very intense magnetic fields^37^, thus enabling new molecular functionalities. Turning to the solution-phase results, our study shows that solvation can lead to dephasing of the electronic dynamics and change the time constants of electronic relaxation. Our dynamical calculations, moreover, show that effects beyond the solvation shifts of the electronic states, such as dissipation and decoherence, are responsible for the observed complete dephasing of the electronic dynamics within 40 fs. Our work, thus, quantifies the effects of complex fluctuating environments under ambient conditions on electronic and vibrational dynamics. Our study demonstrates a general approach to unravelling the impact of solvation on conical intersection dynamics^14^, which also includes the predicted creation of electronic coherences at conical intersections^46^, and to exploring the applicability of the concepts of charge-directed reactivity^47,48^ on solution-phase dynamics.

## Methods

### Experimental set-up

The primary light source used in this experiment was a Ti:sapphire regenerative amplifier providing 4 mJ pulses that were subsequently amplified in a cryogenically cooled two-pass amplifier. The two-stage Ti:sapphire-based laser system provided 17 mJ, ~30 fs pulses at 1 kHz repetition rate, and 90% of the output was used to pump a barium borate-based type II optical parametric amplifier seeded with a white-light supercontinuum originating from the same pump pulse. The parametric amplifier delivered ~1.6 mJ, ~30 fs idler pulses centred at a wavelength of *λ* = 1.8 μm that were passively carrier-envelope-phase stable. This mid-infrared output was then compressed down to sub-three-cycle pulses in a dual-filamentation set-up to ~12 fs (ref. ^15^). The compressed pulses were then focused by a *f* = 250 mm spherical mirror into a high-pressure He gas cell where the broad-band soft-X-ray probe was generated and focused by a toroidal mirror with a focal spot size of ~62 μm onto the submicrometre-thin liquid flat jet. The passing beam was then diffracted from a flat-field variable-line-spacing grating onto a two-dimensional CCD soft-X-ray camera (Andor). The energy resolution of the soft-X-ray variable-line-spacing spectrometer was around 0.3 and 0.4 eV at the carbon and nitrogen K-edges, respectively^49^. The other 10% of the initial 800 nm beam was frequency upconverted in two consecutive BBO crystals to create 266 nm pump pulses. The pump pulses were characterized using a self-diffraction frequency-resolved optical gating device, which provided a measured pulse length of around ~30 fs. This pulse dominated the overall experimental time resolution. The travel distance of the pump pulse was matched with the beam path length of the probe, and a high-precision delay stage was used to vary the time delay between the two pulses. Two 100 μm Ti filters were used to filter out the mid-infrared beam from the soft-X-ray beam and also for differential pumping between the spectrometer and the experimental interaction chamber. Further details of the experimental set-up can be found in ref. ^50^. For both gaseous and aqueous phase measurements, the error bars are the measured standard deviations over 12 and nine sets of scans, respectively, each consisting of 20 spectra for each time delay and every spectra having an exposure time of 4 s (at a laser repetition rate of 1 kHz).

### Sample-delivery systems

To generate the submicrometre-thin liquid flat jet, we used two cylindrical jets with an orifice diameter of ~20 μm and let them collide with each other under an angle of 48°. The home-built sample-delivery device allowed us to produce thicknesses of a few hundreds of nanometres, as our measurements showed^51^. A more detailed description of the flat-jet system and its properties can be found in refs. ^51,52^. Aqueous solutions of 5 M pyrazine were used for the solution-phase measurements and were freshly prepared each day. For the gas-phase experiments, a heating device evaporated the crystalline pyrazine samples into the gas phase at ~51 °C, just below its melting point. This was done in the experimental chamber to avoid condensation on the way. The gas sample was then guided into a metallic cuvette with a thickness of approximately 1 mm.

### Theoretical methods

To assign the peaks in the experimental spectra, electronic structure calculations were conducted at the level of regularized multi-state restricted-active-space second-order perturbation theory^53^ RASPT2/RAS2(10e,8o) for both the initial and final states of the core excitations. The population dynamics was investigated by a non-adiabatic dynamics simulation in the FSSH scheme based on ADC(2)/aug-cc-pVDZ. Time-resolved differential absorbance spectra were constructed using the nuclear ensemble approach by calculating XAS spectra for an ensemble of geometries sampled from FSSH trajectories.

In the RASPT2 calculations, the RAS2 space consisted of the two *n*_N_, three *π* and three π^*^ orbitals. The RAS1 space contained all the carbon and nitrogen 1*s* orbitals in the spectral simulations of the carbon and nitrogen K-edges, respectively. The RAS1 space was fully occupied in the initial states (ground state or valence excited states). The final core-excited states were obtained by enforcing single-electron occupation in RAS1 using the HEXS projection technique available in OpenMOLCAS^31^, and thus, the molecular orbitals in the core-excited states were optimized in the presence of a core hole in RAS1. In the solution-phase calculations, the effects of the solvent were considered by embedding the pyrazine molecule in the PCM. The peak assignments based on RASPT2 were confirmed by calculating the core-excitation energies and oscillator strengths with CC3 and CCSDT.

The 170 and 187 initial conditions of the FSSH simulation were randomly prepared in the 4.56–4.92 eV energy window, which corresponded to the low-energy part of the second absorption band in UV–visible absorption, in the gas and aqueous-solution phases, respectively. The effects of the solvent were considered by including two explicit water molecules and COSMO in the aqueous-solution phase. Each trajectory was propagated with a time step of 0.5 fs up to 200 fs.

Further details of the calculations, including a more exhaustive list of references to the methods used, are given in the Supplementary Information.

## Online content

Any methods, additional references, Nature Portfolio reporting summaries, source data, extended data, supplementary information, acknowledgements, peer review information; details of author contributions and competing interests; and statements of data and code availability are available at 10.1038/s41567-024-02703-w.

## Supplementary information

Supplementary Information Supplementary Figs. 1–29, Tables 1 and 2, further transient-absorption data, theoretical methods and an interpretation of the effects of the solvent.

## Data Availability

The datasets generated or analysed during the current study are available from the corresponding author on reasonable request.
