# Supplementary material for: Electronic dynamics created at conical intersections and its dephasing in aqueous solution
Source: Nat Phys. 2024 Nov 27;21(1):137–45. doi: 10.1038/s41567-024-02703-w (PMC11746140; doi:10.1038/s41567-024-02703-w)
Supplement: Supplementary file 1 — Supplementary Figs. 1–29, Tables 1 and 2, further transient-absorption data, theoretical methods and an interpretation of the effects of the solvent. [file 41567_2024_2703_MOESM1_ESM.pdf]

nature physics

Supplementary information https://doi.org/10.1038/s41567-024-02703-w

Electronic dynamics created at conical intersections and its dephasing in aqueous solution

In the format provided by the authors and unedited

Supporting Information for Electronic dynamics created at conical intersections and its dephasing in aqueous solution

Yi-Ping Chang[1,2†], Tadas Balciunas[1,3†], Zhong Yin[3,4*†], Marin Sapunar[5†], Bruno N. C. Tenorio[6,7†], Alexander C. Paul[8†], Shota Tsuru[9,10], Henrik Koch[8*], Jean–Pierre Wolf[1*], Sonia Coriani[6*] and Hans Jakob Wörner[3*]

[1]GAP–Biophotonics, Université de Genève, 1205 Geneva, Switzerland.
[2]European XFEL, 22689 Schenefeld, Germany.
[3]Laboratory of Physical Chemistry, ETH Zürich, 8093 Zürich, Switzerland.
[4]International Center for SynchrotronRadiation Innovation Smart, Tohoku University, 980-8577 Sendai, Japan.
[5]Division of Physical Chemistry, Ruđer Bošković Institute, 10000 Zagreb, Croatia.
[6]Department of Chemistry, Technical University of Denmark, 2800 Kongens Lyngby, Denmark.
[7]Instituto Madrileño de Estudios Avanzados en Nanociencia, IMDEA-Nanociencia, 28049 Madrid, Spain.
[8]Department of Chemistry, Norwegian University of Science and Technology, 7034 Trondheim, Norway.
[9]Lehrstuhl für Theoretische Chemie, Ruhr-Universität Bochum, 44801 Bochum, Germany.
[10]RIKEN Center for Computational Science, RIKEN, 650-0047 Kobe, Japan.

*Corresponding author(s). E-mail(s): yinz@tohoku.ac.jp; henrik.koch@ntnu.no; jean-pierre.wolf@unige.ch; soco@kemi.dtu.dk; hwoerner@ethz.ch;
†These authors contributed equally to this work.

Contents

1 Additional Transient-Absorption Data **2**

1.1 Time-dependence of differential absorbance at the nitrogen K-edge . . 3

1.2 Depletion-corrected spectra 3

1.3 Pump-probe scans extending to delays of 2 ps 3

2 Theoretical Methods **9**

2.1 Symmetry analysis at the FC geometry (D_{2h} symmetry) 11

2.2 RASPT2 calculations 13

2.3 CCSD and CC3 calculations 14

2.4 Comparison of static CC3 and RASPT2 spectra with experiment at the Carbon and Nitrogen K-edges 20

2.5 Nuclear dynamics 22

2.5.1 Simulated UV spectra 22

2.5.2 Fewest-Switches Surface Hopping calculations 23

2.5.3 Comparing FSSH and MCTDH calculations 24

2.6 Simulated TR-XAS 28

3 Interpretation of the solvent effects **31**

3.1 Concentration-dependent absorption spectra 31

3.2 Self-association effect 32

3.3 Solvent effects in each excited state 34

1 Additional Transient-Absorption Data

Time-dependence of differential absorbance at the carbon K-edge

The time dependence of these carbon K-edge differential absorbance bands is shown in Fig. S1. In gaseous pyrazine, the 281.3-eV band is fitted with a convolution of the gaussian instrument-response function with an exponential decay function $e^{-t/\tau}$ and the 282.3-eV band with a sigmoidal function, as shown in Fig. S1A. The 281.3-eV band, associated with the $^1\mathrm{B}_{2\mathrm{u}}(\pi\pi^*)$ character, has a time constant of 120 ± 20 fs. The 282.3 eV band, which is associated with mixed $^1\mathrm{B}_{3\mathrm{u}}(\mathrm{n}\pi^*)$ and $^1\mathrm{A}_{\mathrm{u}}(\mathrm{n}\pi^*)$ character, has a rise time of 70 ± 20 fs.

The 284.2-eV and 287.2-eV bands (Fig. S1C) are both fitted with sigmoidal functions convoluted with cosines. The 284.2-eV band, which is associated with the $^1\mathrm{A}_{\mathrm{u}}(\mathrm{n}\pi^*)$ character, has a rise-time of 130 ± 40 fs with a time constant of 70 ± 20 fs in the first 150 fs. The 287.2-eV band, which is associated with the $^1\mathrm{B}_{3\mathrm{u}}(\mathrm{n}\pi^*)$ character, has a rise-time of 90 ± 40 fs with a time constant of 50 ± 20 fs, and emerges earlier than the 284.2-eV band by 20 ± 10 fs. The two bands also show weak out-of-phase oscillations.

In aqueous pyrazine, again the 281.3 eV is fitted with a convolution of the gaussian instrument response function with an exponential decay function $e^{-t/\tau}$ and the 281.9 eV band with a sigmoid function, as shown in Fig. S1B. The 281.3 eV band, which is associated with the $^1\mathrm{B}_{2\mathrm{u}}(\pi\pi^*)$ character, has a time constant of 130 ± 40 fs.

The 281.9 eV band, which is associated with mixed $^1B_{3u}(n\pi^*)$ and $^1A_u(n\pi^*)$ character, has a rise time of 50 ± 20 fs. For the 284.4 and 286.8 eV bands (Fig. S1D), they are fitted with a sigmoidal function and an IRF convoluted with an exponential decay , respectively. The 284.4 eV band, which is associated with the $^1A_u(n\pi^*)$ character, has a rise-time of 70 ± 10 fs in the first 100 fs but starts declining in intensity afterward. The 286.8 eV band, which is associated with the $^1B_{3u}(n\pi^*)$ character, has a time constant of 140 ± 40 fs.

1.1 Time-dependence of differential absorbance at the nitrogen K-edge

Looking below the nitrogen pre-edge in gas phase, we have the polynomial fits of 395.3, 396.4 and 397.6 eV bands in Fig. S2A, with character $^1B_{3u}(n\pi^*)$, $^1A_u(n\pi^*)$ and $^1B_{3u}(n\pi^*)$, respectively. These below-edge bands show weaker but still qualitatively comparable oscillations compared to the above-edge bands, showing a contrast in ΔOD intensities near $\sim$90 fs. The weaker oscillations is likely due to a greater overlap of transition peaks below the pre-edge than above the pre-edge (Fig. 2B in the manuscript). Averaging all three of these bands together and fitting them with a sigmoid gives a rise time of 70 ± 40 fs.

Above the nitrogen pre-edge in aqueous pyrazine in Fig. S2B, the 397.8 eV and 400.3 eV bands, which are associated with the $^1B_{3u}(n\pi^*)$ and $^1A_u(n\pi^*)$ characters, respectively, do not display any visible oscillations. From sigmoidal function fits, the 397.8 and 400.3 eV bands have rise times of 90 ± 20 fs and 90 ± 50 fs, respectively.

1.2 Depletion-corrected spectra

This section presents depletion-corrected spectra at the carbon K-edge (Fig. S3) and the nitrogen K-edge (Fig. S4). These spectra were obtained by adding the ground-state spectra (blue shaded areas) scaled by the excitation fraction to the measured ΔOD spectra at positive time delays. The excitation fraction was determined in each measurement by applying a positivity constraint to the depletion corrected spectra.

1.3 Pump-probe scans extending to delays of 2 ps

Measurements were also performed for the initial 2 ps delay range. These are shown in Fig. S5 with mostly similar bands to the initial 150 fs measurements.

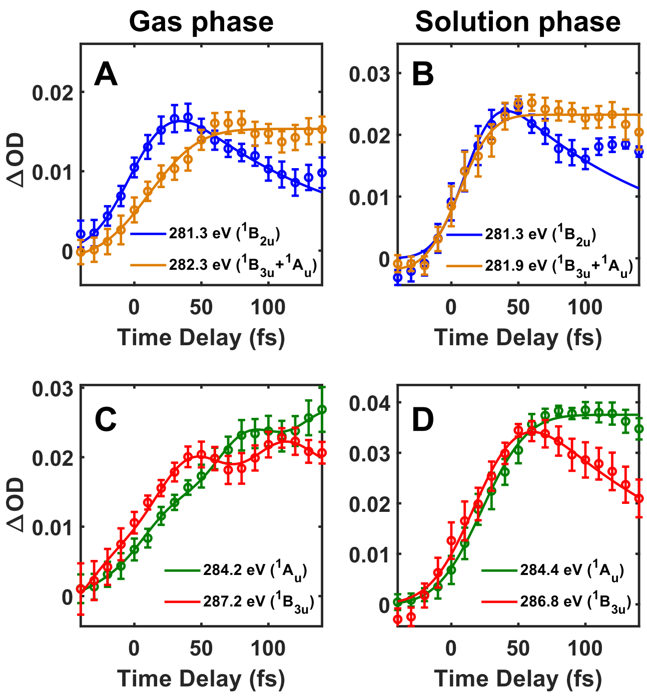

Fig. S1: Time dependence of the differential absorbance bands at the carbon K-edge. **(A)** Lineouts at the carbon K-edge of gaseous pyrazine for the 281.3 and 282.3 eV bands. According to calculations shown in Fig. S3A, the 281.3 band is associated with the $^1B_{2u}$ $(\pi\pi^*)$ character, and the 282.3 eV band with mixed $^1B_{3u}(n\pi^*)$ and $^1A_u(n\pi^*)$ characters, respectively. **(B)** Lineouts at the carbon K-edge of 5M aqueous pyrazine for 281.3 and 281.9 eV bands. According to calculations shown in Fig. S3B, the 281.3 band is associated with the $^1B_{2u}(\pi\pi^*)$ character, and the 281.9 eV band with mixed $^1B_{3u}(n\pi^*)$ and $^1A_u(n\pi^*)$ characters, respectively. **(C)** Lineouts at the carbon K-edge of gaseous pyrazine for 284.2 and 287.2 eV bands. According to calculations shown in Fig. S3A, the 284.2 and 287.2 eV bands are associated with the $^1A_u(n\pi^*)$ and $^1B_{3u}(n\pi^*)$ characters, respectively. The 284.2 eV band has an earlier rise-time than the 287.2 eV band and the two bands show weak out-of-phase oscillations. **(D)** Lineouts at the carbon K-edge of 5M aqueous pyrazine for 284.4 and 286.8 eV bands. According to calculations shown in Fig. S3B, the 284.4 eV band is associated with the $^1A_u(n\pi^*)$ character, while the 286.8 eV band is associated with the $^1B_{3u}(n\pi^*)$ character.

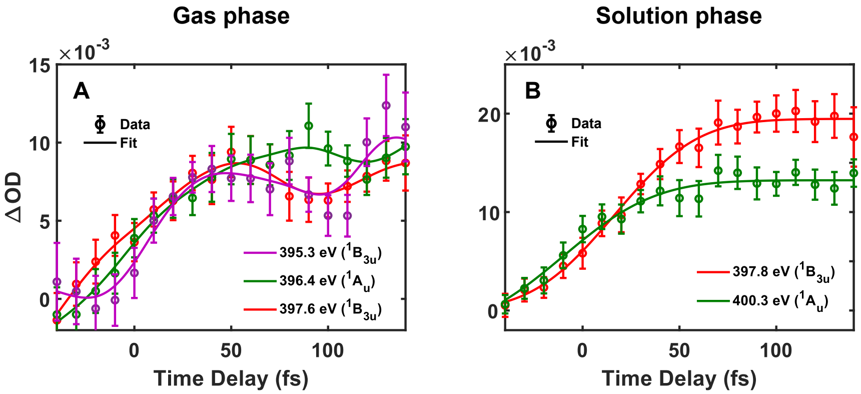

Fig. S2: **(A,B)** Time dependence of differential absorbance bands at the nitrogen K-edge over 150 fs. **(A)** Lineouts at the nitrogen K-edge of gaseous pyrazine for 395.3, 396.4 and 397.6 eV bands. According to calculations shown in Fig. S4A, the 395.3, 396.4 and 397.6 eV bands are associated with the $^1B_{3u}(n\pi^*)$, $^1A_u(n\pi^*)$ and $^1B_{3u}(n\pi^*)$ characters, respectively. **(B)** Lineouts at the nitrogen K-edge of 5M aqueous pyrazine for 397.8 eV and 400.3 eV bands. According to calculations shown in Fig. S4B, the 397.8 eV and 400.3 eV bands are associated with the $^1B_{3u}(n\pi^*)$ and $^1A_u(n\pi^*)$ characters, respectively.

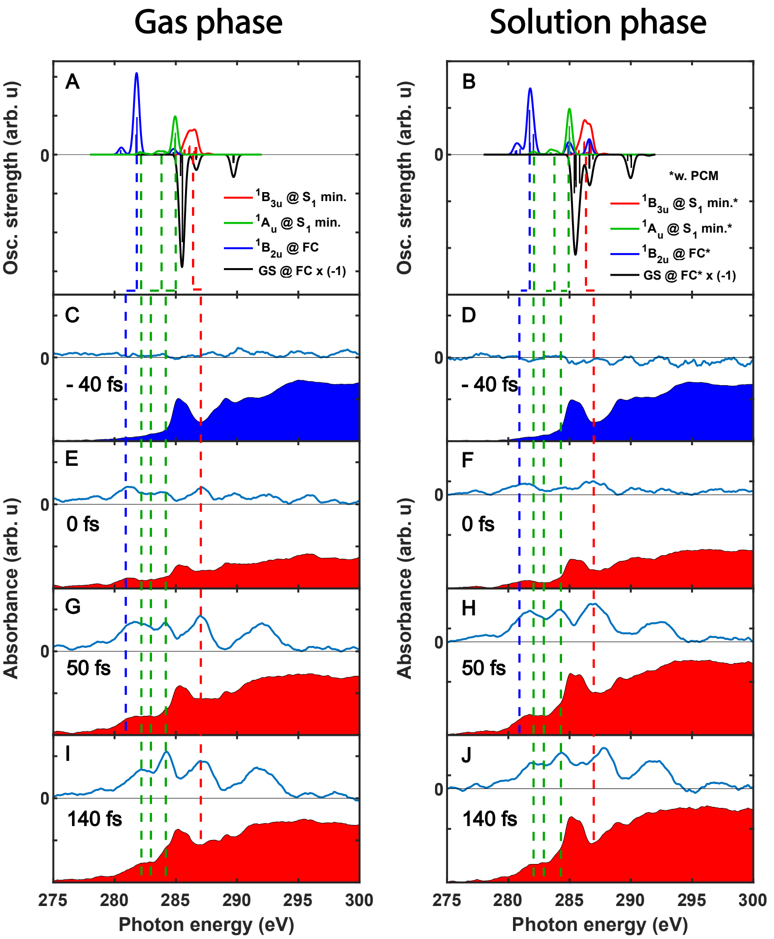

Fig. S3: **(A)**,**(B)** carbon K-edge excited-state XA spectra calculated at the RASPT2/RAS2(10e,8o) level at both the FC and relaxed geometries for the first valence-excited state (S_1), with and without PCM, respectively. Differential absorbance spectra (blue lines) and ground state depletion-corrected spectra (shaded areas) at the carbon K-edge of gaseous pyrazine (**(C)**,**(E)**,**(G)**,**(I)**) and 5M aqueous pyrazine (**(D)**,**(F)**,**(H)**,**(J)**) at different time delays of −40 fs, 0 fs, 50 fs, and 140 fs. The pure ground state spectra are shown by the blue-shaded areas at −40 fs and the depletion-corrected spectra by red-shaded areas at later delays.

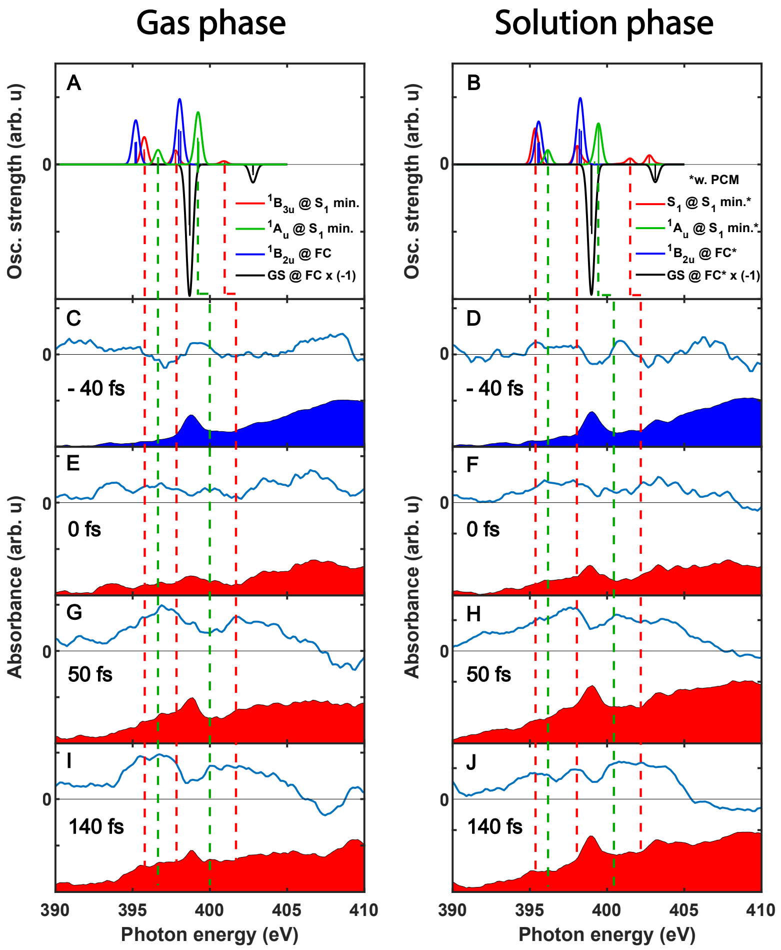

Fig. S4: **(A)**,**(B)** Nitrogen K-edge excited-state XA spectra calculated at the RASPT2/RAS2(10e,8o) level at both the FC and relaxed geometries for the first valence excited state (S_1), with and without PCM, respectively. Differential absorbance spectra (blue lines) and ground state depletion-corrected spectra (shaded areas) at the nitrogen K-edge of gaseous pyrazine (**(C)**,**(E)**,**(G)**,**(I)**) and 5M aqueous pyrazine (**(D)**,**(F)**,**(H)**,**(J)**) at different time delays of −40 fs, 0 fs, 50 fs and 140 fs. The pure ground state spectra are shown by the blue-shaded areas at −40 fs and the depletion-corrected spectra by red-shaded areas at later delays.

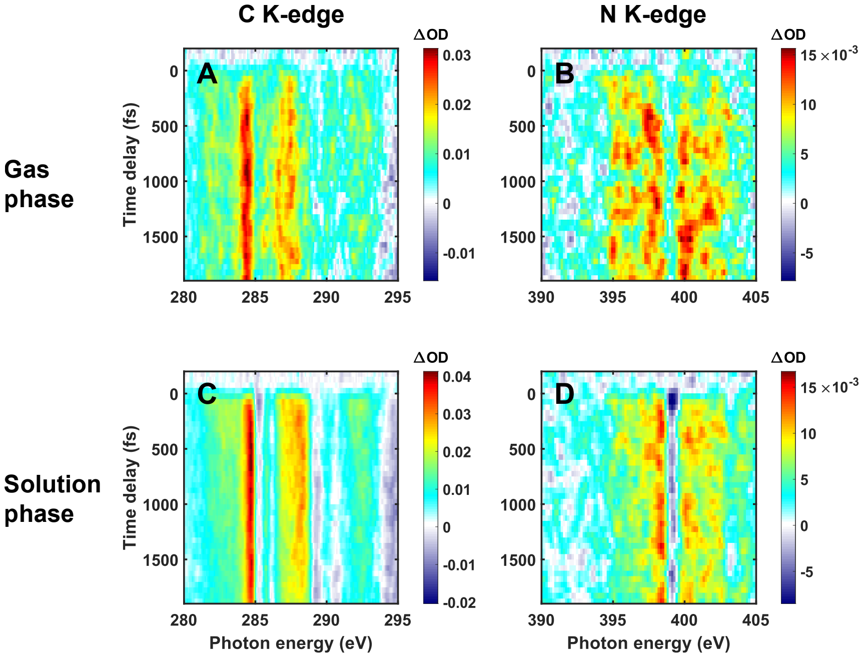

Fig. S5: Time-resolved differential absorbance spectra of pyrazine over the initial $\sim$2 ps. **(A,B)** Time-resolved differential absorbance spectra at the carbon and nitrogen K-edges of gaseous pyrazine, respectively. **(C,D)** Time-resolved differential absorbance spectra at the carbon and nitrogen K-edges of 5M aqueous pyrazine, respectively. These solution-phase data have been recorded with a $\sim$2 times higher pump intensity compared to the data shown in Fig. 2G,H of the main text.

2 Theoretical Methods

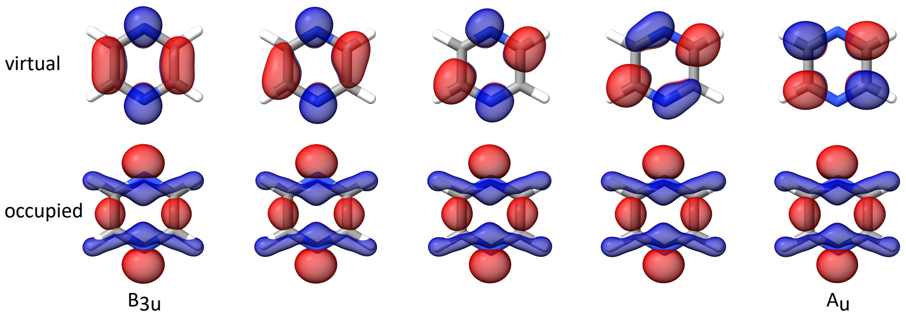

Fig. S6: NTOs for the S_1 state at geometries along a path from a geometry where S_1 can be assigned to B_{3u} to a geometry where the S_1 state can be assigned to A_u symmetry. The geometries used are part of a PES scan utilized to prepare Fig. 4. The scan is a path around the conical intersection as indicated by the arrows on the S_1 surface in Fig. 4. The potential is symmetric and therefore completing the cycle is just mirroring the plot, as shown in Fig. 4 of the main text.

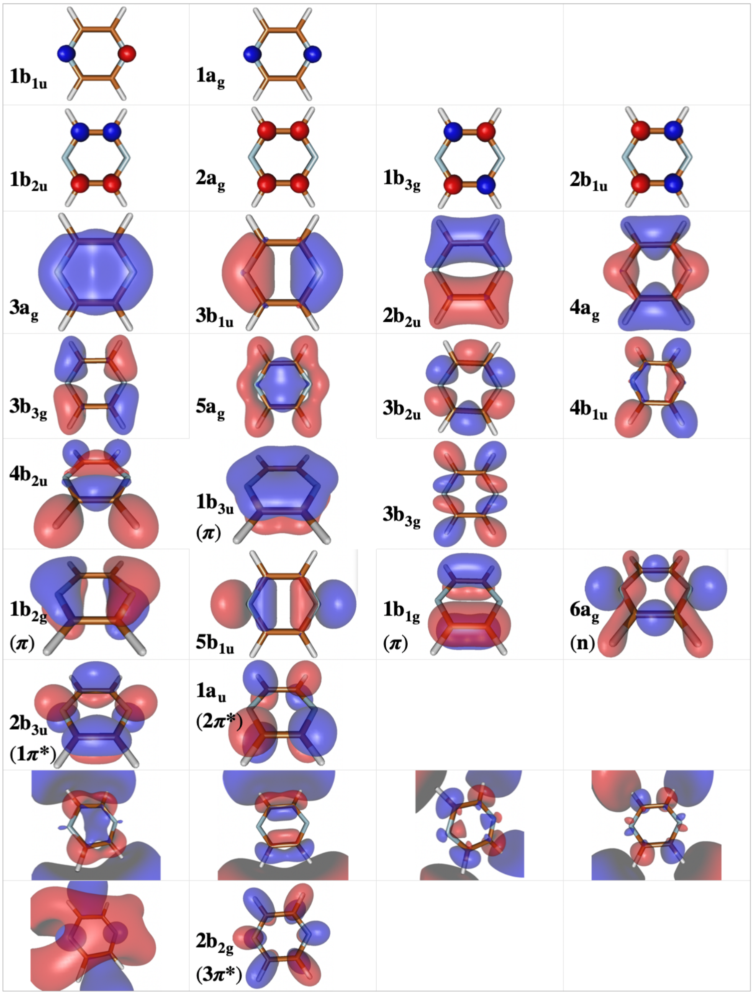

Fig. S7: Occupied molecular orbitals and first virtual orbitals of pyrazine (CAMB3LYP/cc-pVTZ). Mulliken symmetry notation.

2.1 Symmetry analysis at the FC geometry (D_{2h} symmetry)

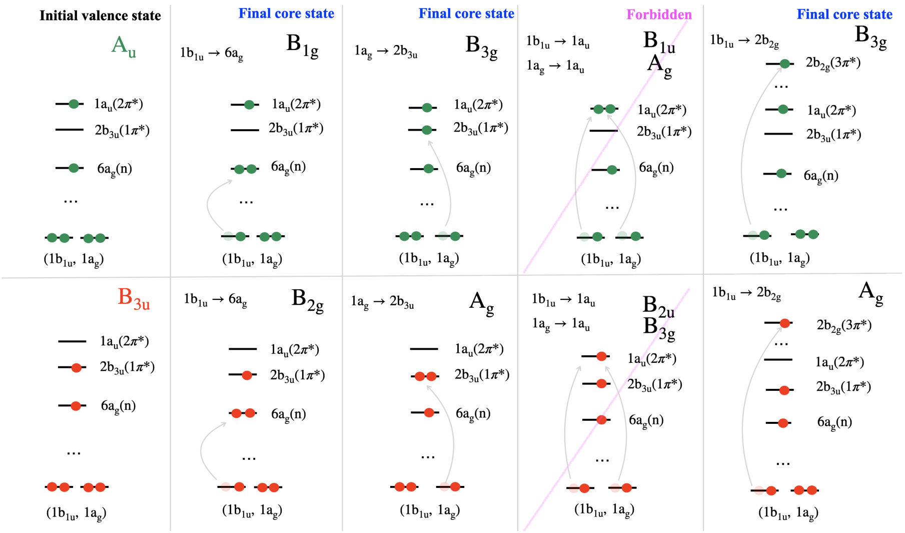

Fig. S8: Schematics of the main (dipole allowed) transitions in the XA spectra of the $^1B_{3u}(n\pi^*)$ and $^1A_u(n\pi^*)$ states at D_{2h} symmetry at the nitrogen K-edge. As indicated, the core excitations of the two states involve the same orbital transitions, but the final symmetry of the core state is different. From ^1A_u, final dipole-allowed core state are B_{1g}, B_{2g} and B_{3g}. From $^1B_{3u}$, final dipole-allowed core state are B_{2g}, B_{1g} and A_g. Hence, transitions from the N 1s orbitals to $1a_u(2\pi^*)$ are dipole forbidden in D_{2h}.

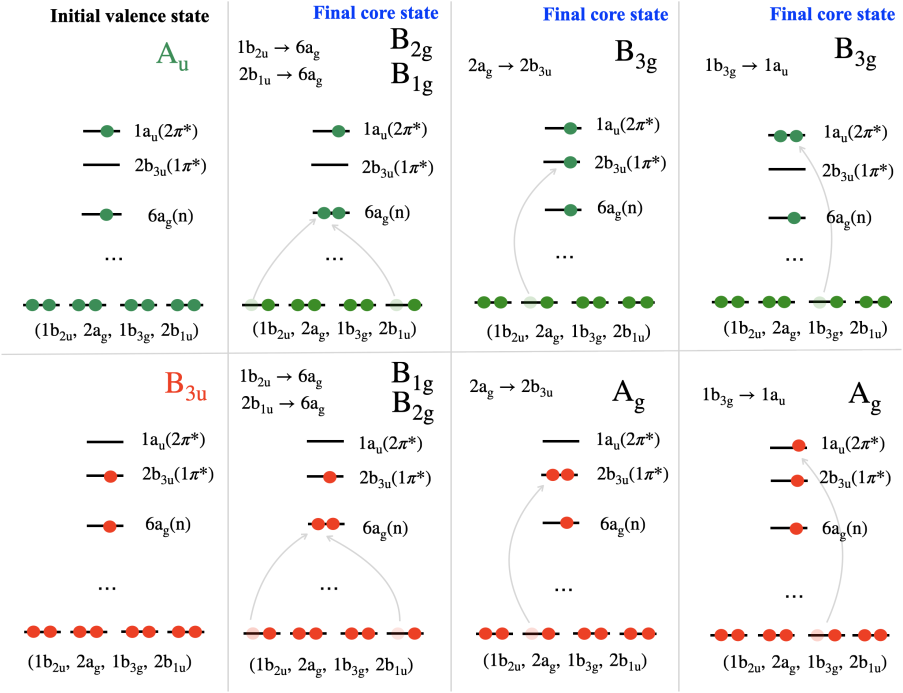

Fig. S9: Schematics of the main transitions in the XA spectra of the $^1B_{3u}(n\pi^*)$ and $^1A_u(n\pi^*)$ states at D_{2h} symmetry at the carbon K-edge. As indicated, the core excitations of the two states involve the same orbital transitions, but the final symmetry of the core state is different. From ^1A_u, final dipole-allowed core state are B_{1g}, B_{2g} and B_{3g}. From $^1B_{3u}$, final dipole-allowed core state are B_{2g}, B_{1g} and A_g.

2.2 RASPT2 calculations

RASPT2 calculations of the X-ray absorption spectra (XAS) of valence-excited states of pyrazine in combination with trajectory-based surface hopping (SH) dynamics performed at the ADC(2) level of theory have been used to simulate the time-resolved X-ray absorption spectra (TR-XAS) at the nitrogen K-edge.

Moreover, we also provide an interpretation of the TR-XAS experiments measured at the nitrogen and carbon K-edges for longer time-delays with relaxed geometries of the S_1 state provided by CASPT2 geometry optimization. The influence of the solvent in the spectra is also investigated with a polarizable continuum model (PCM). For calculations at the nitrogen K-edge, we used the Roos Augmented Double Zeta ANO basis set [1] with the contraction [*4s3p2d*] for N and the ANO-L-DZVP basis set for C and H. For the carbon K-edge calculations, we used the ANO-L-DZVP basis set on all atoms.

Core-excited states were computed by placing the pertinent core orbitals in the RAS1 space and enforcing single electron occupation in it using the HEXS projection technique [2] available in OpenMolcas (*30*), which corresponds to applying the core-valence separation [3]. RAS2 was used for complete electron distribution, i.e., to define the complete active space for the valence electrons while RAS3 was kept empty. We used the same active space described previously by Northey et al. [4]: the 1s orbitals of interest in the RAS1 subspace (two orbitals in case of N and four orbitals in case of C) and eight valence orbitals and ten electrons in the RAS2 subspace (two n_N lone-pairs, three π and three π^*). Throughout, we will refer to this active space with the short notation RASPT2/RAS2(10e,8o). The active orbitals computed at the Franck–Condon (FC) geometry are schematically represented in Fig. S10.

Dynamical correlation effects are further included in the reference space using the regularized multi-state restricted-active-space perturbation theory of the second-order (RMS-RASPT2) approach [5]. An imaginary level shift of 0.25 Hartree was applied to avoid intruder-state singularities. IPEA shift has not been used. For the nitrogen K-edge calculations, initial valence and final core-excited states were obtained by state averaging over 5 and 15 states, respectively. For the carbon K-edge calculations, we state-averaged the same number of initial and 25 final core-excited states.

The ground-state XA spectra at the carbon and nitrogen K-edges computed at the RASPT2/RAS2(10e,8o) level along with the natural transition orbitals (NTOs) of the main transitions are shown in Fig. S11. The choice of active space containing three π^* orbitals and no σ^* or Rydberg orbitals in the active space means that transitions to the latter orbitals cannot be described at the current level of theory. Therefore, some peaks are missing in the simulated spectrum, but the peaks that are present match very well with the experiment.

The spectra computed with the equilibrium geometry of the ground state (FC) and the equilibrium geometry of the first excited state (S_1), both in vacuum, are shown in Fig. S12. The same but using the PCM to mimic solvent effects on the liquid phase are shown in Fig. S13.

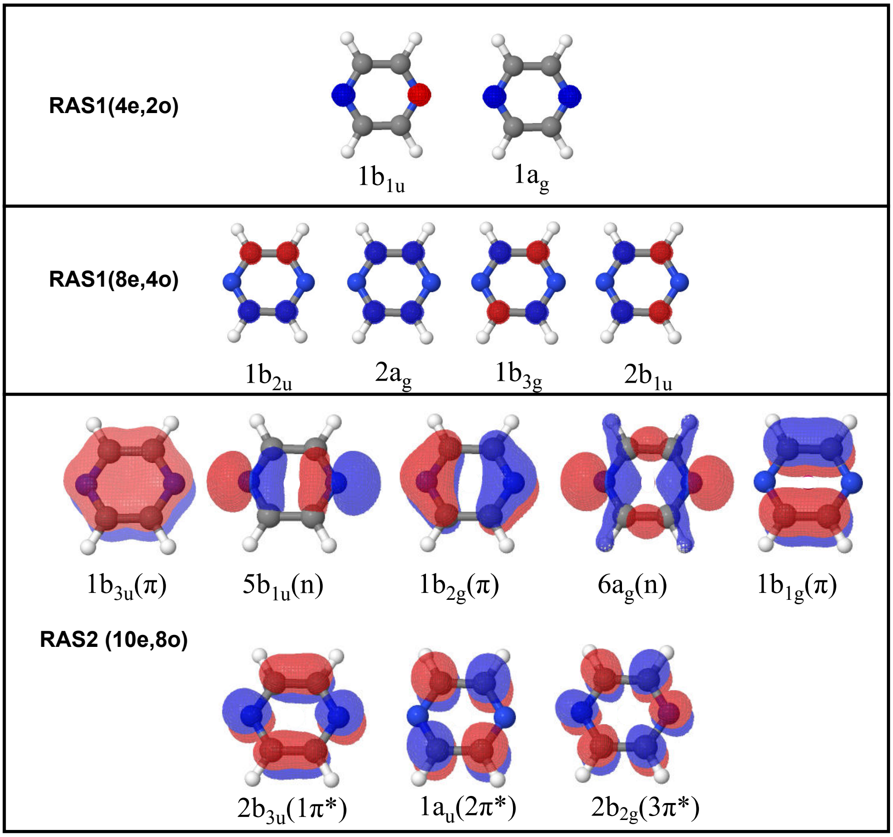

Fig. S10: RASSCF active spaces. RAS1(4e,2o) + RAS2(10e,8o) are used for the nitrogen K-edge calculations, while RAS1(8e,4o) + RAS2(10e,8o) are used for the carbon K-edge calculations.

One advantage of using the RASPT2 approach for TR-XAS is that it can reproduce all orbital configurations, such as $1h1p$, $2h2p$, $3h3p$, etc, within the RAS2 subspace. As long as the selected active space is large enough to account for all important orbital configurations, which is barely guaranteed. On the other hand, the RASPT2 approach has some disadvantages when compared to other single reference methods. It tends to be computationally expensive and time-consuming. Additionally, the accuracy of the calculations strongly relies on the choice of the active space and the number of states included in the state average.

2.3 CCSD and CC3 calculations

Calculations on pyrazine in gas-phase using CCSD and CC3 were performed with the eT program (*33, 34*). Core-valence separation [6] is employed to obtain core-excited states. To ensure that the valence-excited states are orthogonal to the core-excited states the inverse projection (i.e. removing the contributions of core orbitals) is used

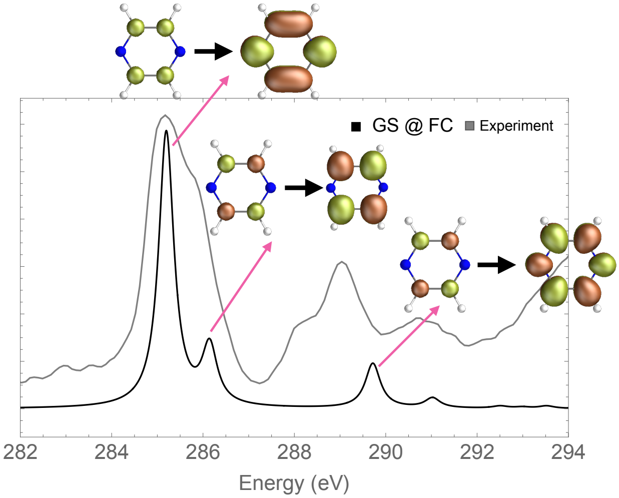

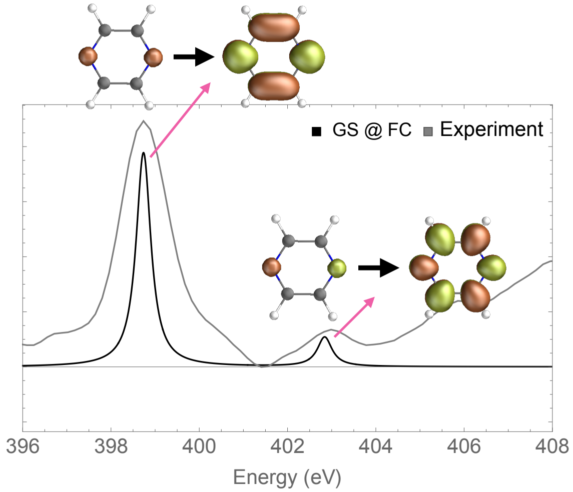

Fig. S11: Ground-state XA spectra (with NTO-based peak-assignments) at the carbon (upper panel) and nitrogen (lower panel) K-edges computed at the RASPT2/RAS2(10e,8o) level versus experiment. Note that no transitions to σ^* are present in the RASPT2 simulated spectra, since these orbitals were not included in the active space.

for the valence excited states. The X-ray absorption spectrum of the ground state at the carbon and nitrogen K-edges along with the NTOs of the main transitions are shown in Fig. S14. For these calculations, the cc-pVDZ basis set plus additional Rydberg-type functions generated according to Kaufmann, Baumeister, and Jungen's prescription [7] was used. The X-ray absorption of the ground and excited states at the Franck-Condon and S_1 minimum geometry was calculated using cc-pVDZ only,

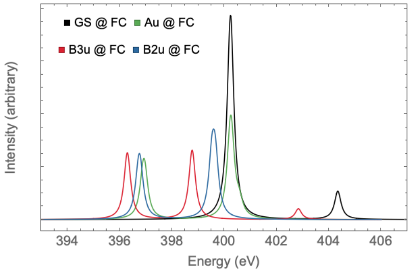

(a) FC nitrogen K-edge

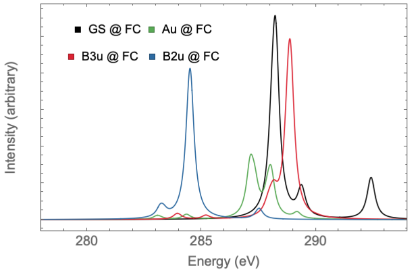

(b) FC carbon K-edge

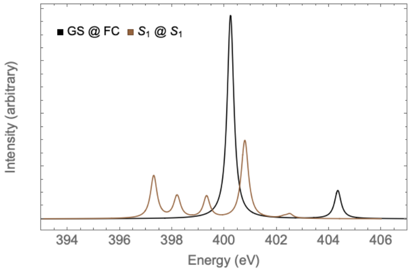

(c) S_1 nitrogen K-edge

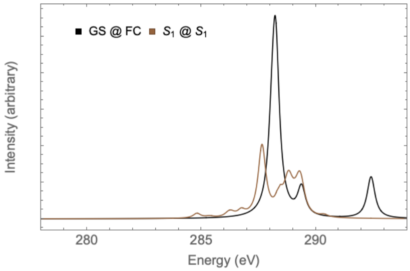

(d) S_1 carbon K-edge

Fig. S12: RASPT2 spectra computed with the equilibrium geometry of the ground state (FC) and the equilibrium geometry of the first excited state (S_1) in vacuum. The S_1 equilibrium geometry was optimized at the CASPT2 level. The computed spectra have been convoluted with Lorentzian functions using HWHM = 0.2 eV. No energy shift has been applied to the calculated energies.

and the spectra are depicted in Fig. S15. Additionally, X-ray absorption spectra were calculated for geometries sampled every 10 fs from 34 ADC(2)/aug-cc-pVDZ SH trajectories using the cc-pVDZ basis set. The resulting false color plot is shown in Fig. S26. In the nitrogen spectra, all peaks have been shifted by -2.72 eV to match the ground state bleach. Peaks predicted to lay above the ground state bleach involve significant double excitation character with respect to the ground state and are thus not described as accurately as the pre-edge peaks at the CC3 level. For this reason, coupled cluster calculations using full triples (CCSDT) and the STO-3G basis have been performed using MRCC [8–10] as a benchmark of the CC3 results. Based on these calculations, an additional shift of -2.6 eV was applied to the core excitations from the valence excited states with an excitation energy larger than the ground state bleach.

In the carbon spectra, all peaks have been shifted by -2.6 eV to match the ground state bleach. Based on CCSDT/STO-3G calculations performed with the eT program (*33*), the excitations from the valence excited states with an excitation energy larger than the ground state bleach have been additionally shifted by -2.0 eV.

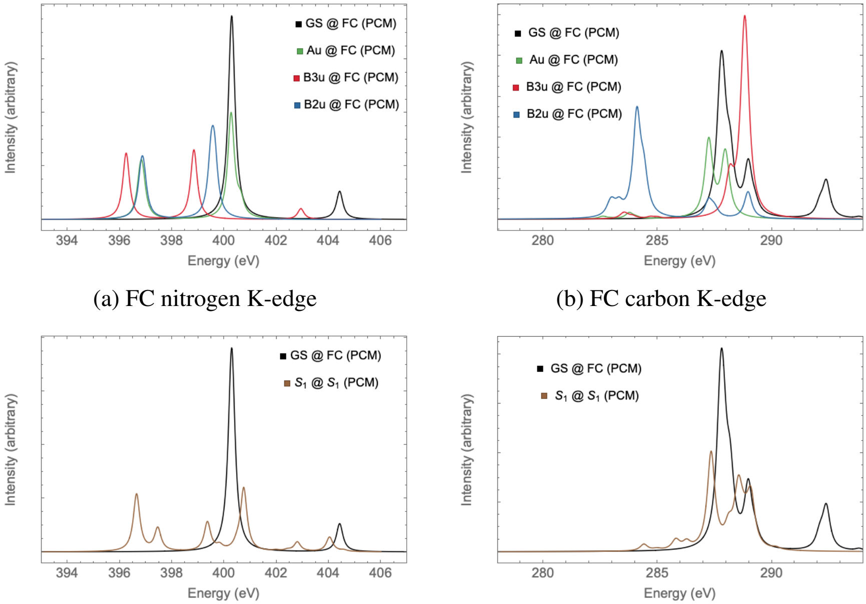

(a) FC nitrogen K-edge

(b) FC carbon K-edge

(c) S_1 nitrogen K-edge

(d) S_1 carbon K-edge

Fig. S13: RASPT2 spectra computed with the equilibrium geometry of the ground state (FC) and the equilibrium geometry of the first excited state (S_1) using a polarizable continuum model (PCM) to mimic solvent effects. The computed spectra have been convoluted with Lorentzian functions using HWHM = 0.2 eV. No energy shift has been applied to the calculated energies.

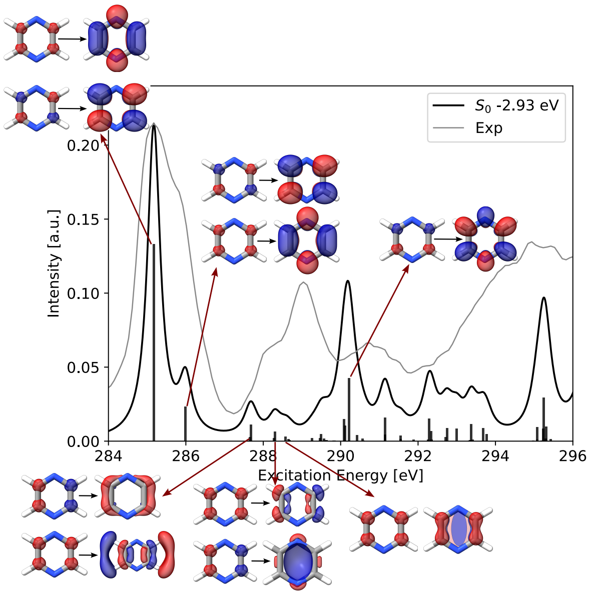

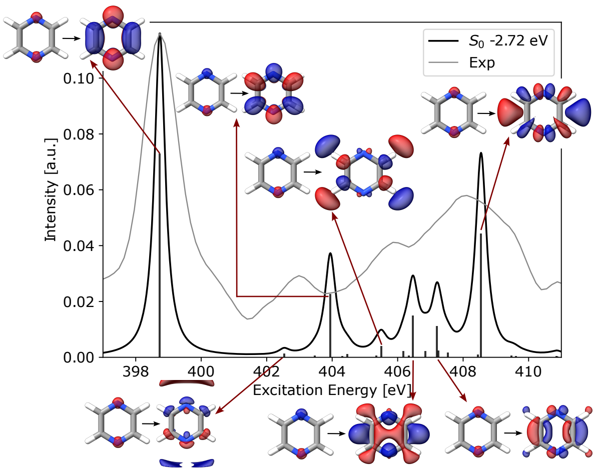

Fig. S14: Ground state XA spectra at the carbon and nitrogen K-edges computed at the CC3 level of theory using the cc-pVDZ basis plus additional Rydberg type functions generated according to Kaufmann, Baumeister and Jungen's prescription [7] with quantum numbers n = 2, 2.5, 3 and angular momentum s and p. NTOs of the main transitions are reported for assignment.

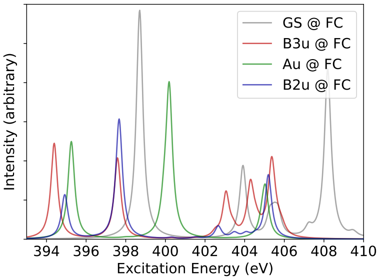

(a) FC nitrogen K-edge

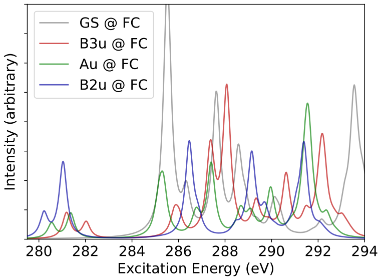

(b) FC carbon K-edge

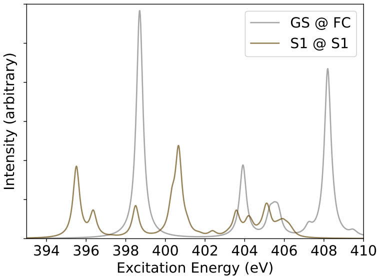

(c) S_1 nitrogen K-edge

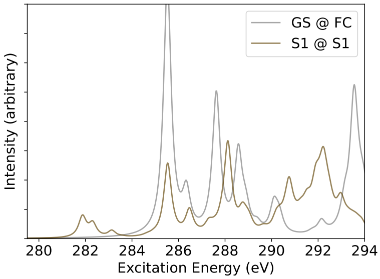

(d) S_1 carbon K-edge

Fig. S15: CC3 spectra computed at the equilibrium geometry of the ground state (FC) and the equilibrium geometry of the first excited state (S_1) in vacuum. The S_1 equilibrium geometry was optimized at the CASPT2 level. The computed spectra have been convoluted with Lorentzian functions using HWHM $= 0.2\,\text{eV}$.

2.4 Comparison of static CC3 and RASPT2 spectra with experiment at the Carbon and Nitrogen K-edges

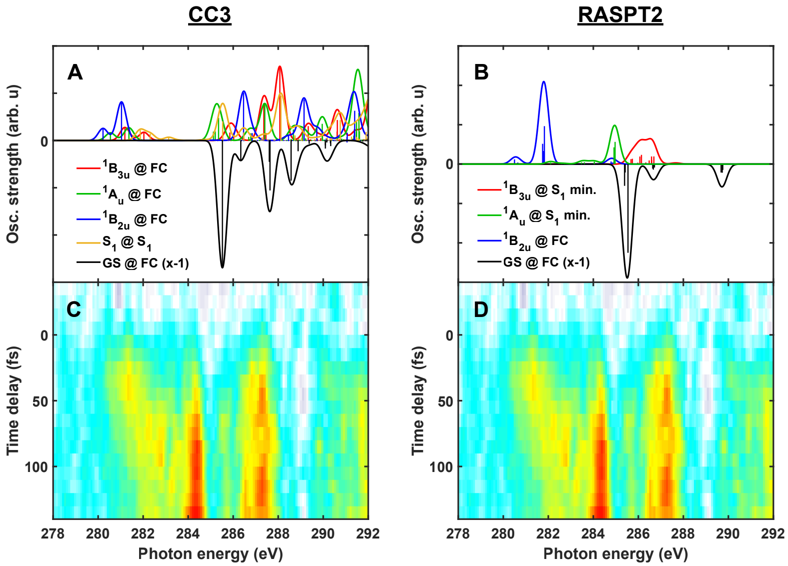

Fig. S16: Carbon K-edge excited-state XA spectra calculated using CC3/cc-pVDZ **(A)** and RASPT2/RAS2(10e,8o) **(B)** at both the FC and relaxed geometries for the first valence excited state (S_1). **(C,D)** Experimental differential absorbance spectra at the carbon K-edge. Note that some transitions are missing in the RASPT2 simulated spectra, since σ^* orbitals were not included in the active space.

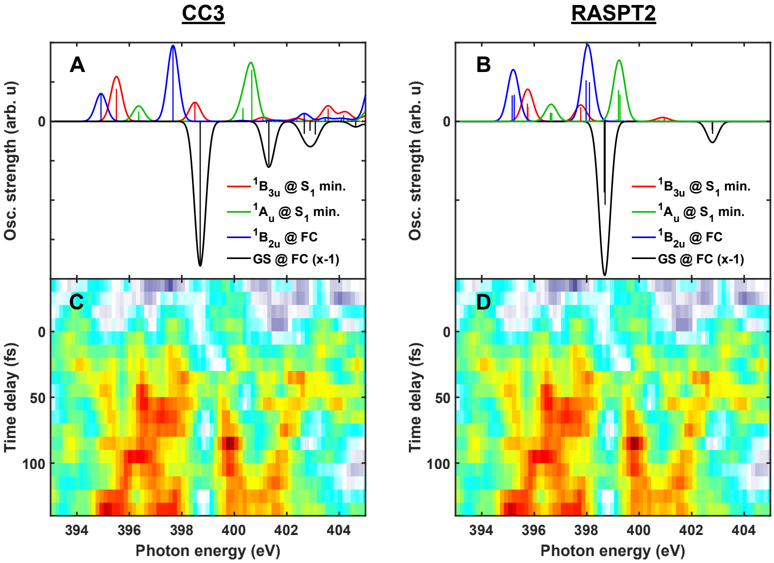

Fig. S17: Nitrogen K-edge excited-state XA spectra calculated using CC3/cc-pVDZ **(A)** and RASPT2/RAS2(10e,8o) **(B)** at both the FC and relaxed geometries for the first valence excited state (S_1). **(C,D)** Experimental differential absorbance spectra at the nitrogen K-edge. Note that some transitions are missing in the RASPT2 simulated spectra, since σ^* orbitals were not included in the active space.

2.5 Nuclear dynamics

2.5.1 Simulated UV spectra

The gas phase and solution UV absorption spectra are shown in Fig. S18a. The spectra are calculated at the ADC(2)/aug-cc-pVDZ level (as implemented in the Turbomole program package [11, 12]) based on an ensemble generated from the harmonic ground state Wigner distribution. For the vacuum spectrum a total of 4000 geometries of pyrazine was sampled. To model the effect of solvation, two water molecules (hydrogen bonded to the nitrogen atoms) were added while the rest of the effect of the environment was modeled implicitly using the conductor like screening model (COSMO) [13, 14]. For this system 2000 initial conditions were sampled from the Wigner distribution with the six lowest frequency normal modes (corresponding to motion of the water molecules relative to pyrazine) frozen as such large amplitude motions are not well described by this approximation.

The first two peaks of the experimental spectrum are reproduced at the ADC(2) level, with a shift of approx. 0.16 eV with respect to the experiment. We see that the solvent shift of the spectrum is correctly reproduced by a combination of two explicit water molecules and COSMO for the bulk environment. Adding either of these effects by itself also resulted in a shift of the spectrum, but by a smaller amount. In Fig. S18b the density of states in the nuclear ensemble is shown, decomposed into contributions from the states of different character. We see that the solvent shifts the two $n\pi^*$ states and the $^1B_{2u}(\pi\pi^*)$ state in opposite directions, with the greatest effect on the $^1A_u(n\pi^*)$ which is shifted above the $^1B_{2u}(\pi\pi^*)$ state at most geometries.

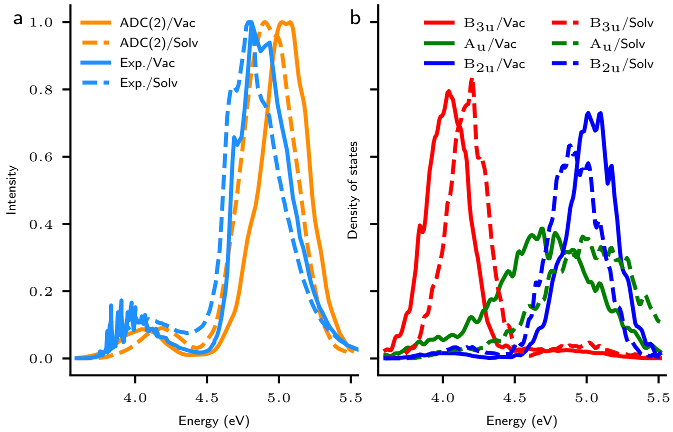

Fig. S18: a) Simulated and experimental UV absorption spectrum of pyrazine in vacuum and in solution. b) Density of states of pyrazine calculated at the ADC(2)/aug-cc-pVDZ level for the ensemble in vacuum and in solution.

2.5.2 Fewest-Switches Surface Hopping calculations

Nonadiabatic dynamics simulations were based on the locally diabatic variant of the fewest-switches surface hopping algorithm (LD-FSSH) with potential energy surfaces (PESs) calculated at the ADC(2)/aug-cc-pVDZ level, following a previous benchmark of the method [15]. This procedure was also successfully used to simulate the time-resolved photoelectron spectrum [16]. A total of 170 initial conditions for nonadiabatic dynamics were randomly (weighted by oscillator strengths) selected from the ground state ensemble among all states falling in an energy window between 4.56 eV and 4.92 eV (excitation window A). This energy window captures the low-energy part of the peak in Fig. S18. Trajectories were propagated for 200 fs using a time step of 0.5 fs. Geometries were sampled from time slices of these trajectories and XA spectra from the currently populated state $L_i(t)$ to a manifold of final core excited states were calculated at the sampled geometries. The TR-XAS spectrum was calculated using the nuclear ensemble approach

$$\sigma(E,t) \propto \sum_i \sum_F \Delta E_{L_i(t)F} \left|\mu_{L_i(t)F}\right|^2 k\big(E - E_{L_i(t)f}, \delta\big)\,, \tag{1}$$

where the sums are over all trajectories i and final states F, ΔE and μ are the (shifted) energy difference and cross sections between the currently populated state $L_i(t)$ and final state F at geometry $R_i(t)$.

For the solution model, an identical computational procedure was employed as in the gas phase to obtain results which are directly comparable. A total of 187 initial conditions were selected for propagation. Hydrogen transfer from the water molecule to the nitrogen atom of pyrazine occurred in one single trajectory. The population dynamics of the remaining trajectories was similar to the one in vacuum. To test the impact of the excitation window on the dynamics, an additional set of (54 in vacuum, 94 in solution) trajectories was calculated with excitation energies between 4.92 eV and 5.5 eV encompassing the higher energy part of the second peak of the UV spectrum of pyrazine (excitation window B).

Analysis of the trajectories and of the potential energy scans was based on projecting the electronic wave functions $\{|\Phi(\mathbf{R})\rangle\}$ calculated at each geometry onto the wave functions of the ground state minimum geometry $\{|\Phi(\mathbf{R}_0)\rangle\}$. The squares of the elements of an orthogonalized matrix of coefficients obtained in this way [16, 17] are then used as a quantitative measure of the electronic character of each state at each geometry described in terms of $^1B_{3u}(n\pi^*)$, $^1A_u(n\pi^*)$, $^1B_{2u}(\pi\pi^*)$ state contributions. Figure S19 shows the diabatic populations in each set of trajectories. The three sets of trajectories share the same qualitative picture of population dynamics. However, we also see some differences in the population oscillations between the ^1A_u and $^1B_{3u}$ states depending on the excitation window. This is especially true at very early times where the first maximum of the $^1B_{3u}$ population seen at higher energies is missing in the lower excitation window. We also see that the population of the ^1A_u is lower in solution than in vacuum.

To support the qualitative description of the dynamics shown in Fig. 4a of the manuscript, we analyze the motion of FSSH trajectories along the 8a and 8b modes in

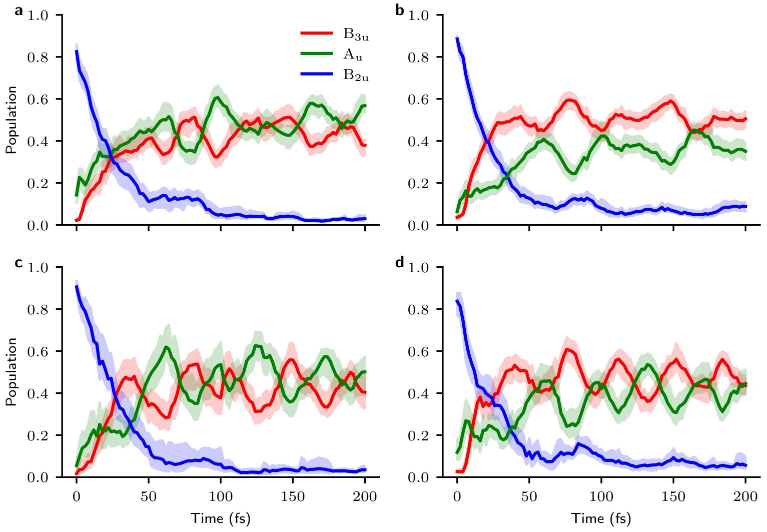

Fig. S19: Diabatic populations along FSSH trajectories of (**a**, **c**) pyrazine excited in excitation windows A and B and (**b**, **d**) pyrazine in solution excited in excitation windows A and B. Shaded areas represent 95% confidence intervals for the population of each state estimated using the bootstrap method.

Fig. S20. The trajectories have been grouped into two sets depending on whether their motion around the CI can primarily be described as clockwise or counterclockwise and the counterclockwise by looking at their motion at each time step with respect to a rolling average representing the central axis at the given time. As expected from symmetry arguments, the number of trajectories falling into each category is approximately the same.

2.5.3 Comparing FSSH and MCTDH calculations

Additional dynamics simulations were performed for the three-state diabatic model Hamiltonian developed by Sala *et al.* [18]. This model includes four electronic states (the ground state and the three lowest excited states) and nine vibrational modes (ν_{6a}, ν_1, ν_{9a}, ν_{8a}, ν_{10a}, ν_4, ν_5, ν_3, ν_{8b}). The nuclear dynamics for this model systems are propagated using FSSH and also the MCTDH method [19, 20] implemented in the Quantics package [21]. For details on the model system and the MCTDH propagation, the reader is referred to Ref. [18]. Populations obtained for the chosen model using FSSH and MCTDH were previously shown to be in good agreement between methods and also with full dimensional ADC(2) calculations [15]. In Fig. S21 we confirm this agreement and also show that the population oscillations disappear when coupling between the $^1\mathrm{A_u}$ and $^1\mathrm{B_{3u}}$ states along the Q_{8b} mode is set to zero.

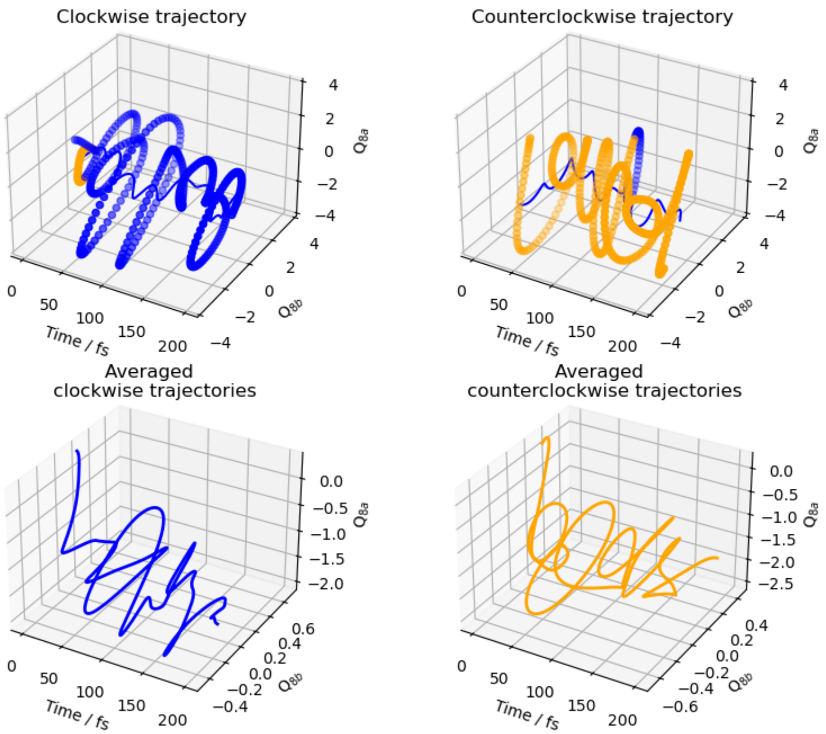

Fig. S20: Dynamics of the FSSH trajectories along the 8a and 8b modes over time. The upper panels show an example trajectory moving primarily clockwise (blue) and counterclockwise (orange) around an average (blue full line). The lower panels show the average dynamics of all trajectories in each group.

In Fig. S22, we show the 2D diabatic reduced density of the wave packet along the 8a and 8b modes. Upon passing through the CI, amplitude is equally sent towards both sides around $Q_{8b} = 0$ on the $^1\mathrm{A_u}$ state. This coincides with a reflection along the 8a mode sending the system back towards the CI. The two components of the wave packet then recombine on the $^1\mathrm{B_{3u}}$ surface on the other side of the CI and the process is repeated. Before 100 fs, a recurrence of the $^1\mathrm{B_{2u}}$ state occurs after which the wave packet is spread out further, but oscillations along the 8a mode along with the spreading + recombining of the wave packet along the 8b mode are still visible.

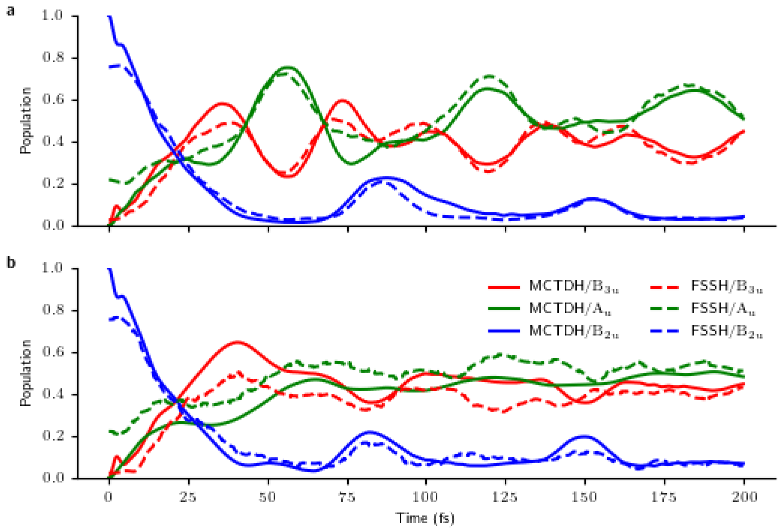

Fig. S21: Comparison of diabatic populations from MCTDH and FSSH calculations performed using (a) the full three-state model from Ref. [18] and (b) the same model, but with coupling between the ^1A_u and $^1B_{3u}$ states along the 8b mode set to zero.

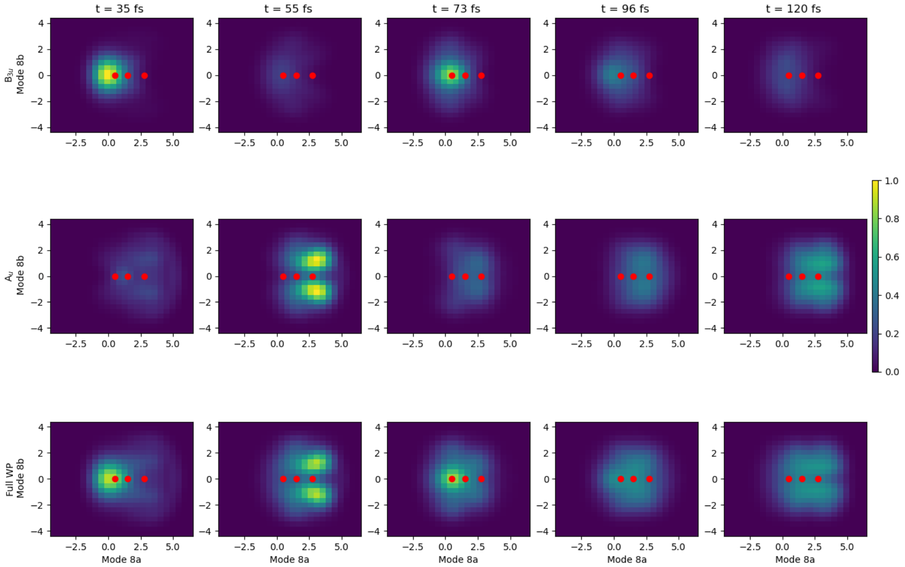

Fig. S22: Motion of the wave packet in the space spanned by the 8a and 8b modes on the potential-energy surfaces from Ref. [18]. Components of the wave packet on the $^1B_{3u}$ (top row) and ^1A_u (middle row) and the two components together (bottom row) are shown. The red dots mark the positions of the $^1B_{3u}$ minimum, the CI and the ^1A_u minimum (from left to right).

2.6 Simulated TR-XAS

Carbon and nitrogen K-edge TR-XAS up to 200 fs were calculated at the RASPT2/RAS2(10e,8o) levels of theory based on time slices taken every 2 fs from 34 of the gas phase trajectories.

Calculating the spectra in this way introduces a discontinuity when sampling geometries from trajectories where the currently populated state is, obviously, calculated at the ADC(2)/aug-cc-pVDZ level and now needs to be re-calculated at a different level of theory. To ensure the consistency of the calculated results, we checked the distribution of excitation energies for all geometries sampled from the dynamics calculated at the ADC(2)/aug-cc-pVDZ, CC3/cc-pVDZ and RASPT2/RAS2(10e,8o) levels of theory. The overall agreement between the three methods is very good, with only a slight difference in the distribution of energies of the S_2 state at the RASPT2 level. However, taking only the subset of geometries at $t = 0$ we see that the agreement between RASPT2 and the other methods is significantly worse, with a significant shift of the S_2 state which is the initially populated state in many trajectories. Despite this difference in the region of the ground state minimum, the energies become much closer at later times, indicating that the shapes of the PES are similar at all three levels. To fix the issue with the different ordering of the states at some geometries at early times, we apply a reordering based on the overlap of the excited state wave functions at the ADC(2) and RASPT2 levels. For the simulated XAS we always choose the RASPT2 state with the largest overlap with the ADC(2) excited state that is populated at the given geometry during the dynamics simulations.

Fig. S23a shows the simulated spectrum at the RASPT2/RAS2(10e,8o) level. The positions of the peaks are accurately represented by the simulated spectrum. Interaction between the two $n\pi^*$ states is clearly visible from the oscillations in the intensity of the peaks. However, these oscillations still have a significantly different time scale and amplitude to the ones seen in the experiment.

As with the nitrogen K-edge, we see a good qualitative agreement with the experimental spectrum. The main difference is the shift of the peaks just below and above the edge in opposite directions, bringing them significantly closer to each other than they are in the experiment.

Fig. S24 shows the integrated intensities of the peaks in the simulated spectra. We see the oscillations and relative intensities of the peaks. These lineouts are highly correlated, but not directly proportional, with the populations of the diabatic states to which the peaks are assigned.

The nitrogen K-edge TR-XAS was also simulated at the CC3/cc-pVDZ level using time slices taken every 10 fs from the same trajectories used for the RASPT2/RAS2(10e,8o) spectra and the same procedure for handling the discontinuity between electronic structure methods for dynamics and spectrum simulations. The state ordering between the ADC(2) and CC3 calculations is significantly more consistent, even at early times close to the CI, than when comparing with RASPT2 calculations.

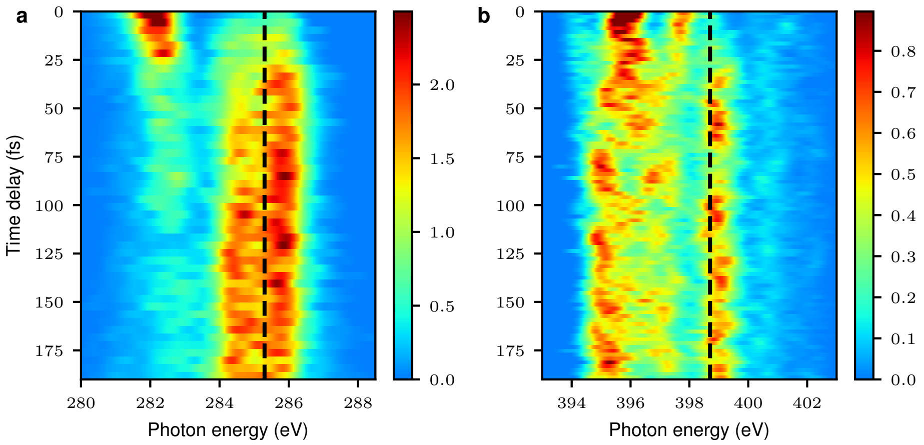

Fig. S23: (**a**) Carbon and (**b**) nitrogen K-edge excited-state XA spectra calculated at the RMS-RASPT2/RAS2(10e,8o) level based on 34 FSSH trajectories calculated at the ADC(2)/aug-cc-pVDZ level. The simulated spectrum has been shifted by -2.9 eV (carbon K-edge) and -1.5 eV (nitrogen K-edge). The position of the ground state bleach is marked by the dashed black line.

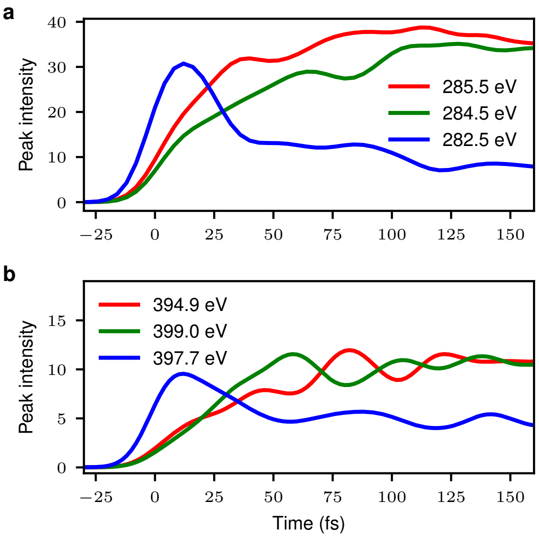

Fig. S24: Integrated intensities of selected peaks in the simulated (**a**) carbon K-edge and (**b**) nitrogen K-edge XA spectra calculated at the RMS-RASPT2/RAS2(10e,8o) level. The red, green and blue lines represent peaks primarily originating from the $^1B_{3u}(n\pi^*)$, $^1A_u(n\pi^*)$ and $^1B_{2u}(\pi\pi^*)$ states, respectively. The full spectrum is shown in Fig. S23.

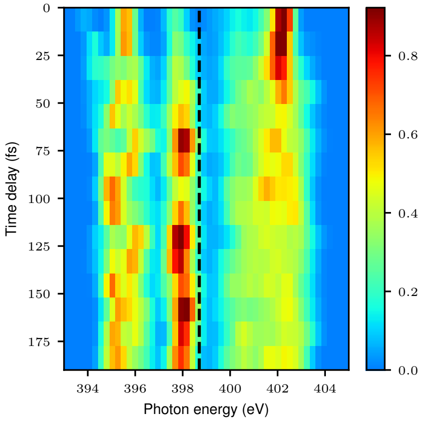

Fig. S25: Nitrogen K-edge excited-state XAS spectra calculated at the CC3/cc-pVDZ level based on 34 FSSH trajectories calculated at the ADC(2)/aug-cc-pVDZ level. The position of the ground state bleach is marked by the dashed black line. The simulated spectrum has been shifted by -2.75 eV with an additional -2.6 eV shift for the above-edge peaks (see Sec 2.3).

3 Interpretation of the solvent effects

3.1 Concentration-dependent absorption spectra

Fig. S26 shows the 266 nm UV pump absorbance of pyrazine solutions across a broad range of concentrations, namely ranging from a 1 mM to 5 M. This was achieved by using various liquid jet thicknesses, down to the sub-μm range [22–24], in order to cover the entire dynamic range. The linear increase in absorbance with pyrazine concentration indicates that self-association does not play a major role on the absorption properties of pyrazine at the studied concentrations. The $\sim$18-μm nozzle orifice size, which was used for 1-5 M linear absorption measurements, was utilized for the time-resolved experiments. The 266 nm source was the same as the one used for the time-resolved experiments.

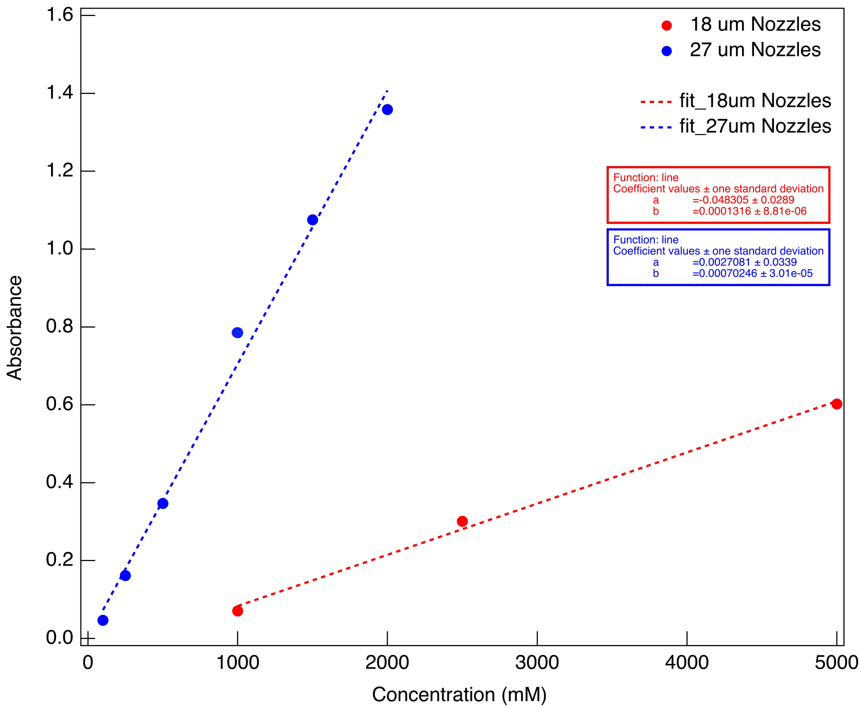

Fig. S26: Absorbance at the pump wavelength (266 nm) by liquid flat jets with different inner diameters of nozzles running aqueous solutions of pyrazine at various concentrations. The linear relationship across the entire concentration range indicates that self-association does not play a major role in the absorption properties of pyrazine at the studied concentrations. The parameters a and b of the linear regressions stand for the intercept and slope, respectively.

Using a standard UV-Vis instrument (Thermo Scientific GENESYS 50), and pyrazine concentrations in the 0.1-2.5 mM range, the extinction coefficient of aqueous pyrazine solutions was found to be ε = 10580 L mol^{-1} cm^{-1}. Using the jet thickness previously measured for $\sim$27 μm nozzle orifice jets [22], the aqueous pyrazine extinction coefficient for jet measurements with these nozzles was extracted via the Beer-Lambert relation and found to be ε_{27} = 8570 L mol^{-1} cm^{-1}, demonstrating reasonable agreement with the value measured from a traditional UV-Vis system. This agreement further suggests that there are no major self-association effects on the absorption properties of aqueous pyrazine even at concentrations above 1 M.

3.2 Self-association effect

The experimental results shown in the previous subsection indicated no major effects of self-association on the UV absorption spectra. A previous study [25] reported small deviations from the Beer-Lambert law over a very broad range (5.5 orders of magnitude) of concentration. In this section, we computationally explored whether insight into self-association of the pyrazine molecules could be obtained from calculations of the UV excitation energies and oscillator strengths of a pyrazine dimer. To this end, we assumed a dimer with a sandwich configuration, where the intermolecular interaction should be the largest, and optimized the geometry at the B3LYP-D3/aug-cc-pVDZ level as in Table S1 and Fig. S27. The optimization yielded as most stable structure a cross-displaced π–π stacked structure, consistent with the CCSD(T) results in Ref. [26]. We then calculated the excitation energies of the dimer at the optimized geometry at the ADC(2)/aug-cc-pVDZ level of theory.

Table S1: Geometry of pyrazine dimer optimized at the B3LYP-D3/aug-cc-pVDZ level of theory; xyz format, coordinates in Angstrom (Å).

C	2.127257	1.134516	-0.323795
C	1.409320	1.133535	0.877978
C	2.127895	-1.133551	-0.325302
C	1.409930	-1.134621	0.876444
H	2.408516	2.074124	-0.803604
H	1.111302	2.071613	1.349250
H	2.409713	-2.072344	-0.806377
H	1.112321	-2.073489	1.346403
N	2.493008	0.001000	-0.933839
N	1.048120	-0.001038	1.487955
C	-2.334762	0.698987	0.706912
C	-2.333932	-0.701496	0.705570
C	-1.201511	0.700790	-1.257781
C	-1.200694	-0.698182	-1.259106
H	-2.799606	1.254518	1.523908
H	-2.798103	-1.259158	1.521499
H	-0.727873	1.258310	-2.067611
H	-0.726384	-1.253564	-2.070013
N	-1.772184	1.409789	-0.275635
N	-1.770503	-1.409732	-0.278336

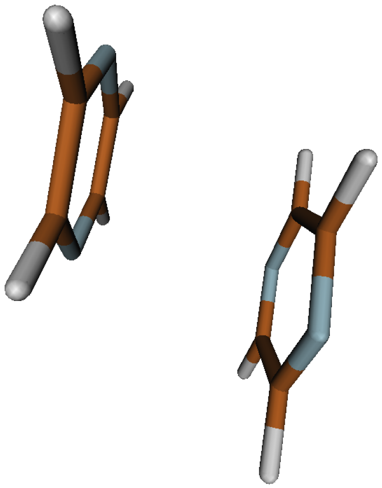

Fig. S27: Visualized geometry of the pyrazine dimer optimized at the B3LYP-D3/aug-cc-pVDZ level.

The energies and oscillator strengths of the excitations of each pyrazine monomer in the dimer and of the dimer as a whole are given in Table S2. NTOs of the dimer excitations are shown in Figure S28.

Table S2: Excited states of each pyrazine monomer in the B3LYP-D3 optimized dimer and of the dimer as a whole, calculated using ADC(2) in conjunction with the aug-cc-pVDZ basis set.

State	Monomer 1 / Monomer 2)		Dimer	
	Exc. energy (eV)	Osc. strength	Exc. energy (eV)	Osc. strength
$^1B_{3u}(n\pi^*)$	4.18153	0.00606	4.16614	0.01371
	4.18085	0.00606	4.17088	0.00153
$^1A_u(n\pi^*)$	4.78947	0.00000	4.75575	0.00000
	4.78909	0.00000	4.77360	0.00000
$^1B_{2u}(\pi\pi^*)$	5.20376	0.09022	5.17327	0.06597
	5.20554	0.09024	5.17526	0.06249

In the dimer, the $^1B_{3u}(n\pi^*)$ and $^1A_u(n\pi^*)$ and $^1B_{2u}(\pi\pi^*)$ pairs of states experience a slight red shift from those of the monomers, although by less than 0.1 eV. While the pair of $^1A_u(n\pi^*)$ states remains dark, the oscillator strengths of the two $^1B_{3u}(n\pi^*)$ states sum up to a value that is slightly larger than twice the strength of the monomer (hyperchromism). For the $^1B_{2u}(\pi\pi^*)$ state, on the other hand, the oscillator strength in the dimer is lower than twice that of the monomer (hypochromism). These results are consistent with the experimental observations.

	Energy (eV)	Osc. Str.	Hole	Particle	Weight
State 1	4.166138	0.013710	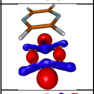	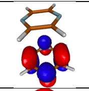	0.69
			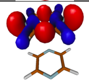	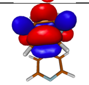	0.16
State 2	4.170876	0.001529	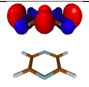	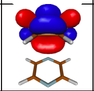	0.69
			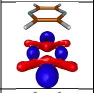	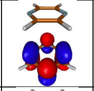	0.16
State 3	4.755755	0.000000	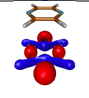	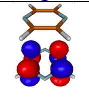	0.83
State 4	4.773597	0.000000	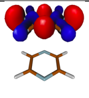	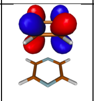	0.83
State 5	5.173270	0.065965	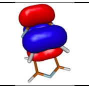	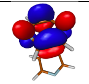	0.70
			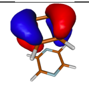	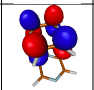	0.12
State 6	5.175263	0.062492	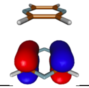	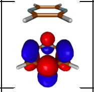	0.70
			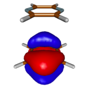	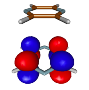	0.12

Fig. S28: Natural transition orbitals of the first 6 valence excited states of pyrazine dimer. ADC(2)/aug-cc-pVDZ results.

3.3 Solvent effects in each excited state

In this section, we study the effects of solvation on pyrazine by building on the spectral simulations based on microsolvation models from Ref. 27. In Ref. 27, 100 snapshots based on an ab initio molecular dynamics (AIMD) simulation for a system where a pyrazine molecule was dissolved in 112 water molecules were obtained. This system corresponds to 0.5 M pyrazine aqueous solution. In each snapshot, the pyrazine molecule and the water molecules in the first and second solvation shells were extracted. The number of extracted water molecules was 24 on average, and the microsolvation model consisting of one pyrazine molecule and 24 water molecules

may qualitatively describe the solvent effects in the present 5M pyrazine aqueous solution, where the unit cell contains one pyrazine and 12 water molecules. For each snapshot of the microsolvation model consisting of the extracted pyrazine and water molecules, the valence excitation energies were calculated at the ADC(2) level, adopting the aug-cc-pVTZ and aug-cc-pVDZ basis sets to the pyrazine and water molecules, respectively. The oscillator strengths for the 100 snapshots were collected and plotted in histogram style in Fig. 5(f) of Ref. 27. Such UV-Vis spectral simulation based on 200 AIMD snapshots was also conducted for an isolated pyrazine molecule and the result was plotted in Fig. 4(a) of Ref. 27.

For one of the 100 snapshots, where the NTOs of the transitions from the ground state to the three lowest adiabatic excited states ($\tilde{\mathrm{A}}$, $\tilde{\mathrm{B}}$ and $\tilde{\mathrm{C}}$) were similar to those at the equilibrium geometry of pyrazine, the solvent effects from the water molecules in the first and second solvation shells were investigated by comparing the excitation energies, the oscillator strengths, and the NTOs for the three models: (i) only the pyrazine molecule, (ii) the pyrazine molecule with the water molecules in the first solvation shell, and (iii) the pyrazine molecule with the water molecules in the first and second solvation shells. The excitation energies and the oscillator strengths were given in the "no-COSMO" columns of Table 3 of Ref. 27. The NTOs were given in Table 4 of Ref. 27. As shown in Table 4 of Ref. 27, without water molecules, $\tilde{\mathrm{A}}$, $\tilde{\mathrm{B}}$ and $\tilde{\mathrm{C}}$ states had almost the pure configuration characters of $^1\mathrm{B}_{3\mathrm{u}}(\mathrm{n}\pi^*)$, $^1\mathrm{A}_\mathrm{u}(\mathrm{n}\pi^*)$ and $^1\mathrm{B}_{2\mathrm{u}}(\pi\pi^*)$, respectively. Energy ordering of the configuration characters of $^1\mathrm{A}_\mathrm{u}(\mathrm{n}\pi^*)$ and $^1\mathrm{B}_{2\mathrm{u}}(\pi\pi^*)$ switched when the water molecules in the second solvation shell were added. Considering the configuration characters in Table 4 of Ref. 27 and the excitation energies given in Table 3 of Ref. 27, it is estimated that the diabatic $^1\mathrm{B}_{3\mathrm{u}}(\mathrm{n}\pi^*)$ and $^1\mathrm{A}_\mathrm{u}(\mathrm{n}\pi^*)$ states are raised by 0.3 and 0.5 eV, respectively, whereas the diabatic $^1\mathrm{B}_{2\mathrm{u}}(\pi\pi^*)$ state is lowered by 0.2 eV, due to the solute-solvent interactions.

The second absorption band in Fig. 4(a) and Fig. 5(f) of Ref. 27 are separated for the adiabatic $\tilde{\mathrm{B}}$ and $\tilde{\mathrm{C}}$ states and shown in panels (a) and (b) of Fig. S29, respectively. The dashed bar corresponds to the central wavelength of the pump pulse of the present experiment, considering the difference between the band maximum of the simulated and experimental spectra shown in Fig. 4(a) of Ref. 27. Since the adiabatic $\tilde{\mathrm{B}}$ state has the configuration character of $^1\mathrm{A}_\mathrm{u}(\mathrm{n}\pi^*)$ at the equilibrium geometry and the excitation from the ground state is dipole forbidden there, contribution from the $\tilde{\mathrm{B}}$ state to the second absorption band in the UV-Vis absorption spectrum of pyrazine in the gas phase is minor, as seen in panel (a) of Fig. S29. Meanwhile as seen in panel (b) of Fig. S29, contributions from the $\tilde{\mathrm{B}}$ and $\tilde{\mathrm{C}}$ states to the second absorption band in the UV-Vis spectrum simulated for the microsolvation model are comparable. The two contributions are almost in equal amount, especially at the energy corresponding to the central wavelength of the experimental pump laser. This behavior is consistent with the switching of the energy ordering observed in Table 4 of Ref. 27 and indicates that a conical intersection between the diabatic $^1\mathrm{A}_\mathrm{u}(\mathrm{n}\pi^*)$ and $^1\mathrm{B}_{2\mathrm{u}}(\pi\pi^*)$ states lies in the FC region.

Normalized frequency distribution of the oscillator strength in the energy window of 4.9–5.1 eV of Fig. S29 (a) and (b) are plotted in Fig. S30 (a) and (b), respectively, to examine whether the $^1\mathrm{A}_\mathrm{u}(\mathrm{n}\pi^*)$ and $^1\mathrm{B}_{2\mathrm{u}}(\pi\pi^*)$ configurations are

noticeably mixed in the FC region. When the target state has the pure $^1\mathrm{A_u}(\mathrm{n}\pi^*)$ or $^1\mathrm{B_{2u}}(\pi\pi^*)$ configuration character, the oscillator strength of the transition from the ground state is 0.00 and 0.10, respectively, at the ADC(2)/aug-cc-pVTZ level. Panel (a) thus indicates that the two configurations are not mixed when a pyrazine molecule is isolated. Meanwhile, panel (b) exhibits evidence of the configuration mixing in the FC region by distribution of the oscillator strength around 0.04 due to the solute-solvent interactions. This configuration mixing may enable faster nonadiabatic transition to $^1\mathrm{A_u}(\mathrm{n}\pi^*)$ or almost direct optical transition with the pump laser to the diabatic $^1\mathrm{A_u}(\mathrm{n}\pi^*)$ state when pyrazine is dissolved in water. This interpretation is consistent with the early appearance of the core-excitation peaks which are assigned to the $^1\mathrm{A_u}(\mathrm{n}\pi^*)$ state in the solution phase (see Figs. 2 and 3 of the main manuscript and Fig. S1 of this SI).

The solvent effects observed in the simulation of Ref. 27 also gives possible interpretation of suppression of the oscillatory flow of population between the $^1\mathrm{B_{3u}}(\mathrm{n}\pi^*)$ and $^1\mathrm{A_u}(\mathrm{n}\pi^*)$ states. The potential energy surface of $^1\mathrm{A_u}(\mathrm{n}\pi^*)$ is raised by 0.5 eV whereas that of $^1\mathrm{B_{3u}}(\mathrm{n}\pi^*)$ is raised by only 0.3 eV in the solution phase (compare $\tilde{\mathrm{A}}$-$\tilde{\mathrm{B}}$ of bare/no-COSMO and $\tilde{\mathrm{A}}$-$\tilde{\mathrm{C}}$ of +second shell/no-COSMO in Table 3 of Ref. 27. The higher the $^1\mathrm{A_u}(\mathrm{n}\pi^*)$ state surface with respect to the $^1\mathrm{B_{3u}}(\mathrm{n}\pi^*)$ state surface, the smaller the area of the $^1\mathrm{B_{3u}}(\mathrm{n}\pi^*)$/$^1\mathrm{A_u}(\mathrm{n}\pi^*)$ conical intersection becomes. Moreover, energy dissipates from the nuclear wave packet of pyrazine dissolved into the water bath. As a result, the transitions between the $^1\mathrm{B_{3u}}(\mathrm{n}\pi^*)$ and $^1\mathrm{A_u}(\mathrm{n}\pi^*)$ states might be prevented, especially in the direction from $^1\mathrm{B_{3u}}(\mathrm{n}\pi^*)$ to $^1\mathrm{A_u}(\mathrm{n}\pi^*)$.

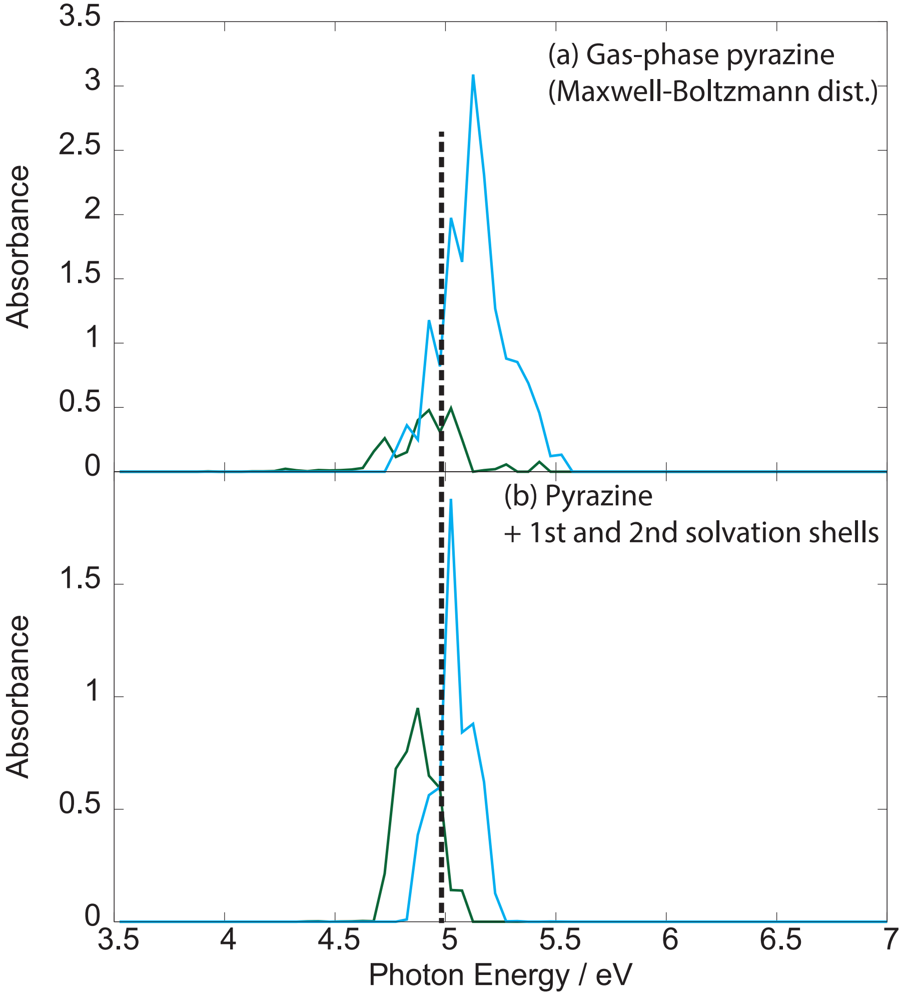

Fig. S29: Absorbances of the electronic transitions from the ground state to the (deep green) $\tilde{\mathrm{B}}$ and (cyan) $\tilde{\mathrm{C}}$ adiabatic states plotted based on the Maxwell-Boltzmann distributions for (a) pyrazine in the gas phase and (b) pyrazine embedded in the first and second solvation shells of water molecules. The excitation energies were calculated with ADC(2) adopting the aug-cc-pVTZ and aug-cc-pVDZ basis sets to the pyrazine and water molecules, respectively. The dashed bar indicates the energy corresponding to the central wavelength of the pump pulse (4.66 + 0.31 eV). These plots are based on computational data for Fig. 4(a) and Fig. 5(f) of Ref. 27.

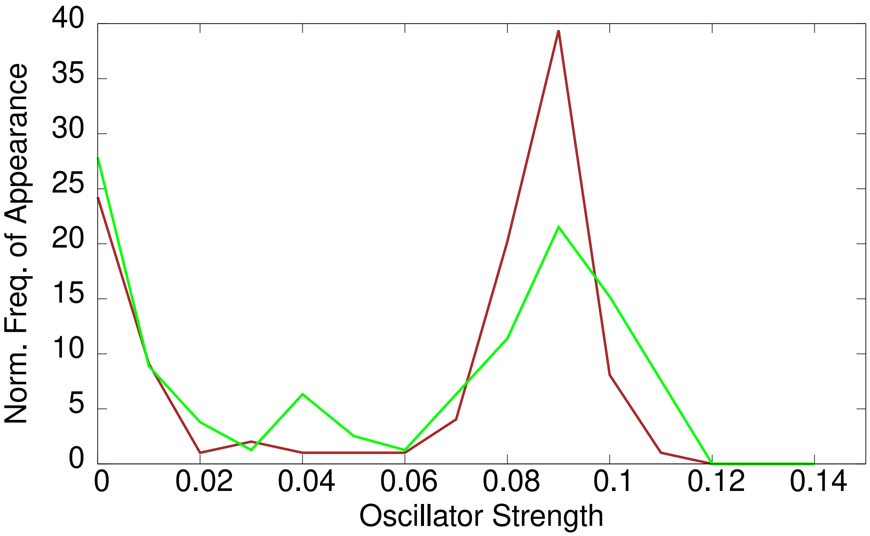

Fig. S30: Normalized frequency distribution of the oscillator strengths in excitation energy of 4.9-5.1 eV calculated for pyrazine (brown) in the gas phase and (green) embedded in the first and second solvation shells of water molecules. The oscillator strengths of the transitions from the ground state to the second and third excited states at the optimized geometry are 0.00 and 0.10, respectively. These plots are based on computational data from Fig. 4(a) and Fig. 5(f) of Ref. 27.

References

[1] Widmark, P.-O., Malmqvist, P.-Å., Roos, B.O.: Density matrix averaged atomic natural orbital (ANO) basis sets for correlated molecular wave functions. Theoretica Chimica Acta **77**(5), 291–306 (1990) https://doi.org/10.1007/BF01120130

[2] Delcey, M.G., Sørensen, L.K., Vacher, M., Couto, R.C., Lundberg, M.: Efficient calculations of a large number of highly excited states for multiconfigurational wavefunctions. J. Comput. Chem. **40**(19), 1789–1799 (2019) https://doi.org/10.1002/jcc.25832

[3] Cederbaum, L.S., Domcke, W., Schirmer, J.: Many-body theory of core holes. Phys. Rev. A **22**, 206–222 (1980)

[4] Northey, T., Norell, J., Fouda, A.E.A., Besley, N.A., Odelius, M., Penfold, T.J.: Ultrafast nonadiabatic dynamics probed by nitrogen K-edge absorption spectroscopy. Phys. Chem. Chem. Phys. **22**, 2667–2676 (2020) https://doi.org/10.1039/C9CP03019K

[5] Battaglia, S., Fransén, L., Fdez. Galván, I., Lindh, R.: Regularized CASPT2: an Intruder-State-Free Approach. J. Chem. Theory Comput. **18**(8), 4814–4825 (2022)

[6] Coriani, S., Koch, H.: Communication: X-ray absorption spectra and core-ionization potentials within a core-valence separated coupled cluster framework. J. Chem. Phys. **143**(18), 181103 (2015) https://doi.org/10.1063/1.4935712

[7] Kaufmann, K., Baumeister, W., Jungen, M.: Universal Gaussian basis sets for an optimum representation of Rydberg and continuum wavefunctions. J. Phys. B: Atom. Mol. Opt. Phys. **22**(14), 2223–2240 (1989) https://doi.org/10.1088/0953-4075/22/14/007

[8] Kállay, M., Nagy, P.R., Mester, D., Rolik, Z., Samu, G., Csontos, J., Csóka, J., Szabó, P.B., Gyevi-Nagy, L., Hégely, B., Ladjánszki, I., Szegedy, L., Ladóczki, B., Petrov, K., Farkas, M., Mezei, P.D., Ganyecz, Á.: The MRCC program system: Accurate quantum chemistry from water to proteins. J. Chem. Phys. **152**(7), 074107 (2020) https://doi.org/10.1063/1.5142048

[9] Kállay, Mihály and Nagy, Péter R. and Mester, Dávid and Gyevi-Nagy, László and Csóka, József and Szabó, P. Bernát and Rolik, Zoltán and Samu, Gyula and Csontos, József and Hégely, Bence and Ganyecz, Ádám and Ladjánszki, István and Szegedy, Lóránt and Ladóczki, Bence and Petrov, Klára and Farkas, Máté and Mezei, Pál D. and Horváth, Réka Anna : MRCC, a quantum chemical program suite. `https://mrcc.hu/`. Accessed: 2023-11-10 (2023)

[10] Kállay, M., Surján, P.R.: Higher excitations in coupled-cluster theory. J. Chem. Phys. **115**(7), 2945–2954 (2001) https://doi.org/10.1063/1.1383290

[11] TURBOMOLE V7.5 2020, a development of University of Karlsruhe and Forschungszentrum Karlsruhe GmbH, 1989-2007, TURBOMOLE GmbH, since 2007; available from
`https://www.turbomole.org`.

[12] Balasubramani, S.G., Chen, G.P., Coriani, S., Diedenhofen, M., Frank, M.S., Franzke, Y.J., Furche, F., Grotjahn, R., Harding, M.E., Hättig, C., Hellweg, A., Helmich-Paris, B., Holzer, C., Huniar, U., Kaupp, M., Marefat Khah, A., Karbalaei Khani, S., Müller, T., Mack, F., Nguyen, B.D., Parker, S.M., Perlt, E., Rappoport, D., Reiter, K., Roy, S., Rückert, M., Schmitz, G., Sierka, M., Tapavicza, E., Tew, D.P., Wüllen, C., Voora, V.K., Weigend, F., Wodyński, A., Yu, J.M.: Turbomole: Modular program suite for *ab initio* quantum-chemical and condensed-matter simulations. J. Chem. Phys. **152**, 184107 (2020) https://doi.org/10.1063/5.0004635

[13] Klamt, A., Schüürmann, G.: Cosmo: a new approach to dielectric screening in solvents with explicit expressions for the screening energy and its gradient. J. Chem. Soc., Perkin Trans. 2, 799–805 (1993) https://doi.org/10.1039/P29930000799

[14] Karbalaei Khani, S., Marefat Khah, A., Hättig, C.: COSMO-RI-ADC(2) excitation energies and excited state gradients. Phys. Chem. Chem. Phys. **20**, 16354–16363 (2018) https://doi.org/10.1039/C8CP00643A

[15] Xie, W., Sapunar, M., Došlić, N., Sala, M., Domcke, W.: Assessing the performance of trajectory surface hopping methods: Ultrafast internal conversion in pyrazine. The Journal of Chemical Physics **150**(15), 154119 (2019)

[16] Piteša, T., Sapunar, M., Ponzi, A., Gelin, M.F., Došlić, N., Domcke, W., Decleva, P.: Combined surface-hopping, dyson orbital, and b-spline approach for the computation of time-resolved photoelectron spectroscopy signals: The internal conversion in pyrazine. Journal of Chemical Theory and Computation **17**(8), 5098–5109 (2021)

[17] Sapunar, M., Piteša, T., Davidović, D., Došlić, N.: Highly Efficient Algorithms for CIS Type Excited State Wave Function Overlaps. J. Chem. Theory Comput. **15**(6), 3461–3469 (2019) https://doi.org/10.1021/acs.jctc.9b00235 . Accessed 2023-03-22

[18] Sala, M., Lasorne, B., Gatti, F., Guérin, S.: The role of the low-lying dark nπ* states in the photophysics of pyrazine: a quantum dynamics study. Physical Chemistry Chemical Physics **16**(30), 15957–15967 (2014)

[19] Beck, M.H., Jäckle, A., Worth, G.A., Meyer, H.-D.: The multiconfiguration

time-dependent Hartree (MCTDH) method: A highly efficient algorithm for propagating wavepackets. Phys. Rep. **324**(1), 1–105 (2000) https://doi.org/10.1016/S0370-1573(99)00047-2

[20] Meyer, H.-D., Manthe, U., Cederbaum, L.S.: The multi-configurational time-dependent Hartree approach. Chem. Phys. Lett. **165**(1), 73–78 (1990) https://doi.org/10.1016/0009-2614(90)87014-I

[21] G. A. Worth, K. Giri, G. W. Richings, I. Burghardt, M. H. Beck, A. Jäckle, and H.-D. Meyer. The QUANTICS Package, Version 1.1, (2015), University of Birmingham, Birmingham, U.K.

[22] Yin, Z., Luu, T.T., Wörner, H.J.: Few-cycle high-harmonic generation in liquids: in-operando thickness measurement of flat microjets. Journal of Physics: Photonics **2**(4), 044007 (2020)

[23] Ekimova, M., Quevedo, W., Faubel, M., Wernet, P., Nibbering, E.T.J.: A liquid flatjet system for solution phase soft-x-ray spectroscopy. Structural Dynamics **2**(5), 054301 (2015) https://doi.org/10.1063/1.4928715

[24] Füle, M., Kovács, A., Gilinger, T., Karnok, M., Gaál, P., Figul, S., Marowsky, G., Osvay, K.: Development of an ultrathin liquid sheet target for laser ion acceleration at high repetition rates in the khz-range. High Power Laser Science and Engineering, 1–24

[25] Peral, F., Gallego, E.: A study by ultraviolet spectroscopy on the self-association of diazines in aqueous solution. Spectrochim. Acta Part A **59**(6), 1223–1237 (2003) https://doi.org/10.1016/S1386-1425(02)00304-9

[26] Feng, J.-Y., Lee, Y.-P., Hsu, P.-J., Kuo, J.-L., Ebata, T.: Structures of (Pyrazine)$_2$ and (Pyrazine)(Benzene) Dimers Investigated with Infrared–Vacuum Ultraviolet Spectroscopy and Quantum-Chemical Calculations: Competition among π–π, CH···π, and CH···N Interactions. J. Phys. Chem. A **127**(19), 4291–4301 (2023) https://doi.org/10.1021/acs.jpca.3c01767 https://arxiv.org/abs/https://doi.org/10.1021/acs.jpca.3c01767

[27] Tsuru, S., Sharma, B., Marx, D., Hättig, C.: Structural sampling and solvation models for the simulation of electronic spectra: Pyrazine as a case study. Journal of Chemical Theory and Computation **19**(8), 2291–2303 (2023) https://doi.org/10.1021/acs.jctc.2c01129
